# Effect of the Gel Drying Method on Properties of Semicrystalline Aerogels Prepared with Different Network Morphologies

**DOI:** 10.3390/gels11060447

**Published:** 2025-06-10

**Authors:** Glenn A. Spiering, Garrett F. Godshall, Robert B. Moore

**Affiliations:** Department of Chemistry, Macromolecules Innovation Institute, Virginia Tech, Blacksburg, VA 24061, USA; gaspiering@vt.edu (G.A.S.); vtechg10@vt.edu (G.F.G.)

**Keywords:** aerogel, xerogel, cryogel, gel drying methods, semicrystalline polymer aerogel, aromatic polymers, X-ray scattering, aerogel morphology, mesoporous adsorbent

## Abstract

The purpose of this study was to investigate the effect of different drying methods on the structure and properties of semicrystalline polymer aerogels. Aerogels, consisting of either globular or strut-like morphologies, were prepared from poly(ether ether ketone) (PEEK) or poly(phenylene sulfide) (PPS) and dried using vacuum drying, freeze-drying, or supercritical CO_2_ extraction. Vacuum drying was found to result in aerogels with a higher shrinkage, smaller mesopores (with pore widths of 2–50 nm), and smaller surface areas compared to the use of supercritical extraction as the drying method. Freeze-dried aerogels tended to have properties between those of vacuum-dried aerogels and aerogels prepared with supercritical extraction. High network connectivity was found to lead to improved gel modulus, which increased the ability of aerogels to resist network deformation due to stresses induced during drying. The PEEK and PPS aerogel networks consisting of highly connected strut-like features were considerably stiffer than those composed of globular features, and thus shrank less under the forces induced by vacuum drying or freeze-drying. The aerogels prepared from PPS were found to have larger mesopores and smaller surface areas than the aerogels prepared from PEEK. The larger mesopores of the PPS aerogels induced lower capillary stresses on the aerogel network, and thus shrank less. This work demonstrates that preparing PEEK and PPS gels with strut-like features can allow aerogel processing with simpler evaporative drying methods rather than the more complex supercritical drying method.

## 1. Introduction

A gel is a system consisting of a substantially dilute solid phase that encompasses a large volume of liquid or gas. Numerous classes of gels exist, including chemical gels and physical gels [1]. While network junctions of a chemical gel are covalent bonds, physical gels rely on physical interactions such as helix formation, ionic interactions, hydrogen bonds, and crystallites to form the network and impart solid-like properties to the gel structure [2,3,4]. Semicrystalline polymer gels are prepared through the dissolution of a semicrystalline polymer in a solvent at elevated temperatures, which is subsequently cooled to a gelation temperature where the polymer is allowed to crystallize, thereby locking multiple polymer chains into a single crystallite. Tie chains link the crystallites together, forming a physical network that spans the entire volume of the gel.

In theory, any semicrystalline polymer can constitute the physical network of a semicrystalline polymer gel. Physical gelation has been reported for numerous semicrystalline polymers, including isotactic polypropylene [5], syndiotactic polystyrene [6], poly(ethylene terephthalate) [7], polyethylene [8], poly(vinyl chloride) [9], and poly(phenylene oxide) [10]. Previously, our group prepared physical gels with numerous semicrystalline polymers, including poly(ether ether ketone) [11,12,13] and poly(phenylene sulfide) [14]. Our previous research found that strut-like aerogel morphologies, resulting from gelation of PEEK or PPS in 1,3-diphenylacetone (DPA) [11,14], efficiently distributed compressive stresses, yielding aerogels with a higher modulus than aerogels consisting of globular morphologies, which were the product of gelation of PEEK in 4-chlorophenol (4CP) or dichloroacetic acid (DCA) [12,13]. PEEK aerogels exhibiting a strut-like morphology appeared to shrink minimally under freeze-drying [11], while PEEK aerogels consisting of a globular morphology were previously found to shrink and densify considerably with freeze-drying when compared to the same aerogels dried through supercritical extraction [13].

Commonly, liquid is removed from a gel network through three primary methods: evaporative drying, yielding gels sometimes referred to as xerogels; freeze-drying, yielding gels sometimes referred to as cryogels; and supercritical extraction, commonly referred to as aerogels [15]. Developments in the field of gel processing have allowed the production of materials with aerogel-like properties through freeze-drying and vacuum drying [15]; as such, we refer to gels dried with all three methods as aerogels.

Evaporative drying, including ambient pressure drying and vacuum drying, is commonly regarded as the most aggressive drying method for gels, and is often avoided in aerogel preparation. However, evaporative drying generally has the most promise for industrial applications, as it has the lowest energy cost and is an easily scalable, well-established technology [16]. Evaporation from fine pores of a gel induces considerable capillary pressure on the gel network. Capillary pressure is calculated as follows:

(1) $$P_{c}=\frac{-2\gamma cos\theta }{r_{c}}$$

where *γ* is the fluid/vapor surface tension of the liquid phase filling the gel pores, *θ* is the contact angle between the pore wall and the fluid/vapor interface, and *r_c_* is the capillary size. The liquid properties, the surface tension, and the contact angle, have considerable influence on the capillary pressure exerted during drying. Pore size also influences the capillary pressure, as capillary pressure increases with decreasing radius. Porous systems consisting of small pores on the nanometer scale are subject to the highest forces [17].

Freeze-drying is generally considered a gentler method for preparing aerogels. While not commonly used for fragile silica aerogels, as monolithic aerogels are not achievable without additional treatment, solvent exchange, or reinforcement [18,19,20], increased development of more robust polymeric systems has led to a more common application of freeze-drying. While freeze-drying itself avoids the strong capillary forces associated with the liquid-to-vapor transition, other undesirable forces induced upon liquid freezing may cause fracture or shrinkage of the bulk gel. The freezing of the pore-filling fluid is depressed with decreasing pore size, as pore size can be smaller than the minimum size required to form stable crystalline nuclei [21]. Depending on the freezing temperature, the liquid within pores on the nanometer length scale may not be able to freeze. If the network matrix is weak, it may be favorable for ice crystals in larger pores to grow, thereby deforming the network [21]. The liquid within small pores supplies the liquid at the crystal growth front, emptying the pores, analogous to capillary forces observed in evaporative drying. While freeze-drying induces less stress on a gel than evaporative drying, it comes at the trade-off of higher energy costs and generally being relegated to a batch process [16].

Supercritical extraction is the most widely used and gentlest method to prepare aerogels. Since extraction near the supercritical point of the pore-filling fluid avoids both the liquid–vapor interface responsible for capillary pressure and the growth of crystals within the porous structure, it can remove the pore-filling fluid with minimal deformation. The energy cost of maintaining the high pressures involved with supercritical drying detracts from its wider application in industry [16]. While supercritical drying, in theory, mitigates forces that can cause shrinkage, in practice, several parameters specific to the process and the sample may result in shrinkage. Condensation of solvent vapors after returning to ambient conditions [22], incomplete solvent extraction [23], the rate of solvent exchange [24], and depressurization rate [24] can induce stresses on the gel, thereby causing shrinkage.

Few studies have investigated the influence of drying techniques on pore morphology and aerogel properties for the same aerogel system dried with evaporative drying, freeze-drying, and supercritical drying. To our knowledge, the research on the effects of the three most common gel drying methods has been carried out on gel networks composed of cellulose [25,26] pectin [27], and crosslinked resorcinol–formaldehyde [28]. Generally, these studies found that while shrinkage was highest for all systems dried with evaporative drying, the lowest shrinkage could occur from either freeze-drying or supercritical extraction, depending on the system and the preparation procedures. While some authors found shrinkage to be comparable between freeze-drying and supercritical drying [28], others found that freeze-drying produced the lowest shrinkage of aerogels [26,27] or that supercritical extraction yielded the lowest shrinkage [25]. In general, however, it is commonly recognized that supercritical drying tends to retain the delicate gel nanostructure [25,26,27], while freezing of ice crystals prior to freeze-drying tends to create large macropores as the ice crystals grow [25,26,27,28]. In addition to the creation of large macropores, ice crystals also tend to compress fine structures of the gel on the nanometer scale [25,26]. Considerable collapse and compression of porosity are observed in aerogels prepared with evaporative drying [25,26,27]. In the literature, aerogel specific surface areas were found to be particularly sensitive to the drying method. Supercritical extraction results in aerogels with the highest surface areas in the range of 200–400 m^2^/g [25,26,27]. In these same studies, freeze-drying tended to result in aerogels with an order-of-magnitude-lower surface area, of 10–60 m^2^/g. Further, these studies highlight that the selection of the solvent is critical for ambient-condition drying. Two of the studies found that evaporative drying from water resulted in negligible surface areas [26,27]. Another study found that ambient pressure drying from isopropanol resulted in aerogels with high surface areas of up to 100 m^2^/g and that drying from ethanol under the same conditions resulted in low surface areas, below 1 m^2^/g [25].

This work reports the preparation of monolithic aerogels composed of strut-like or globular morphologies prepared using poly(ether ether ketone) (PEEK) or poly(phenylene sulfide) (PPS) as the matrix material and dried using either vacuum drying, freeze-drying, or supercritical drying. Building on our previous findings that aerogels consisting of strut-like morphologies are stronger than aerogels consisting of globular morphologies [11] and are thus more resistant to the forces induced during drying, this work expands the scope of previous studies by also comparing vacuum drying, which is conveniently available but subjects samples to high stresses. We hypothesize that fibrillar aerogels, due to their impressive mechanical properties, should be strong enough to resist the forces of capillary pressure induced during vacuum drying and not shrink considerably. To our knowledge, this is the first work to specifically investigate the effects of gel morphology on shrinkage caused by the forces induced during vacuum drying, freeze-drying, and supercritical extraction on a synthetic semicrystalline polymer system. A morphological analysis, including scanning electron microscopy and X-ray scattering, was performed to investigate the effects of drying on the morphology of the semicrystalline network. Aerogel shrinkage, density, and porosity were investigated to observe the effects of densification caused by drying on bulk aerogel properties. The mesopore size (where mesopores are defined as pores with widths of 2–50 nm), mesopore volume, and surface area were found for each aerogel system in order to understand the effects of the drying method on the pore structure of aerogels. Finally, the mechanical properties of the aerogels were determined and used in a model to compare the maximum stress that can be supported by the network to the pressure induced during vacuum drying. The broader impact of this work is to highlight the importance of network connectivity and compressive modulus in resisting collapse during drying in semicrystalline polymer aerogels.

## 2. Results and Discussion

### 2.1. Aerogel Morphology 

**Figure 1:**
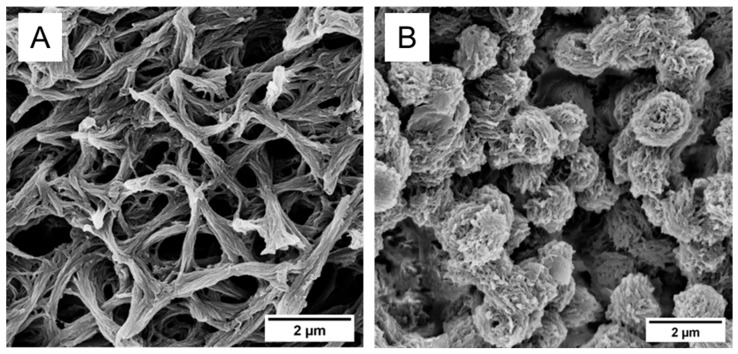
Comparison of the internal morphology of aerogels consisting of (**A**) a strut-like morphology and (**B**) a globular morphology.

Previously, our group found that mechanically robust, monolithic PEEK aerogels can be created using a thermally induced phase separation (TIPS) process with solutions in 4CP or a more benign solvent, DPA, followed by solvent extraction. Figure 1 compares the solvent-extracted morphologies of PEEK aerogels prepared from DPA and 4CP. With a strut-like morphology (Figure 1A), we found these aerogels to be significantly more resilient to the forces exerted during freeze-drying than the globular PEEK aerogels (Figure 1B) prepared using DCA or 4CP as the gelation solvent [11]. We also previously reported strut-like PPS aerogels resulting from the TIPS gelation of PPS in DPA, and these aerogels are also mechanically resistant to densification during freeze-drying [14]. In order to understand the effects of the drying method (i.e., vacuum drying, freeze-drying, or supercritical CO_2_ drying) on a solid PEEK or PPS network, aerogel morphology was probed with SEM and SAXS.

**Figure 2:**
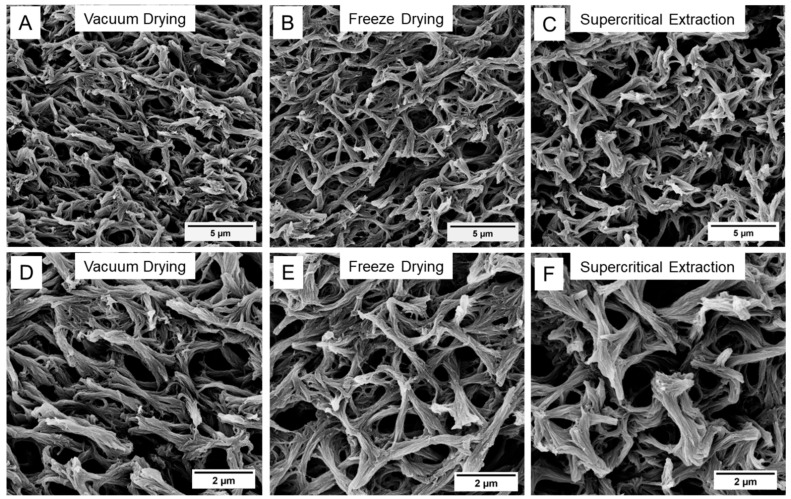
SEM micrographs of the PEEK aerogels gelled from a 13.0 vol% PEEK/DPA solution and dried with (**A**,**D**) vacuum drying, (**B**,**E**) freeze-drying, or (**C**,**F**) supercritical extraction. Images were collected at (**A**–**C**) 10 k× magnification or (**D**–**F**) 20 k× magnification.

SEM images recorded for the PEEK aerogels prepared from a 13.0 vol% PEEK/DPA solution and dried with vacuum drying, freeze-drying, or supercritical extraction can be found in Figure 2. SEM images recorded for the PEEK aerogels prepared from an 8.6 vol% or a 17.5 vol% PEEK/DPA solution and dried with the different drying methods can be found in Figures S2 and S3, respectively. All the PEEK aerogels prepared from DPA displayed a strut-like morphology consisting of polymer axialites, in agreement with our previous work [11]. These PEEK aerogels displayed subtle differences in axialite size and surface texture, when compared across the different drying techniques, as observable by SEM. While the splayed ends of the axialites appeared wider for the samples prepared with supercritical extraction, the samples prepared with vacuum drying or freeze-drying appeared to collapse and be narrower, with a denser texture. Macropore texture largely appears unaffected by the drying method.

**Figure 3:**
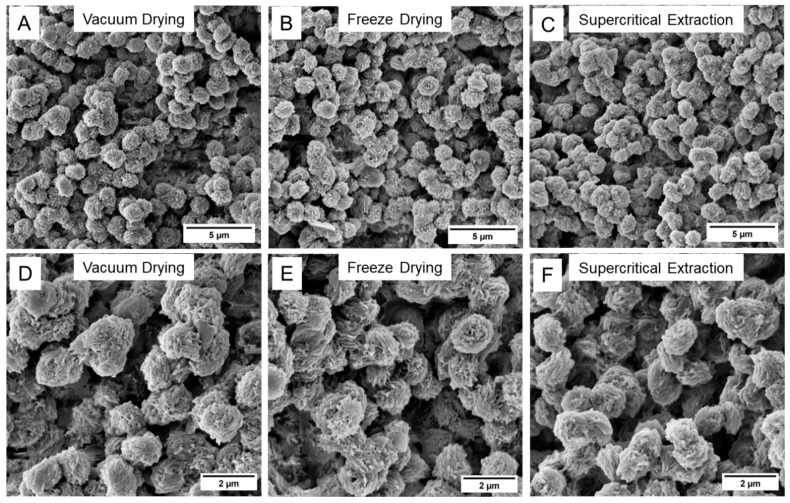
SEM micrographs of the PEEK aerogels gelled from a 13.0 vol% PEEK/4CP solution and dried with (**A**,**D**) vacuum drying, (**B**,**E**) freeze-drying, or (**C**,**F**) supercritical extraction. Images were collected at (**A**–**C**) 10 k× magnification or (**D**–**F**) 20 k× magnification.

Figure 3 shows SEM images recorded for the PEEK aerogel prepared from a 13.0 vol% PEEK/4CP solution and dried with vacuum drying, freeze-drying, or supercritical extraction. SEM images obtained on the PEEK aerogels prepared from an 8.6 vol% or a 17.5 vol% PEEK/4CP solution and dried with vacuum drying, freeze-drying, or supercritical extraction can be found in Figure S4 or Figure S5, respectively. In distinct contrast to the PEEK/DPA aerogels, the PEEK aerogels prepared from 4CP displayed a globular, apparently spherical morphology. All the PEEK aerogels gelled in 4CP displayed minimal differences in aggregate size and surface texture when compared across the different drying techniques, as observable by SEM. The aerogels prepared from 17.5 vol% PEEK/4CP solutions (Figure S4) displayed a considerably smaller particle size compared to the aerogels prepared at 8.6 vol% (Figure S5) or 13.0 vol% (Figure 3).

**Figure 4:**
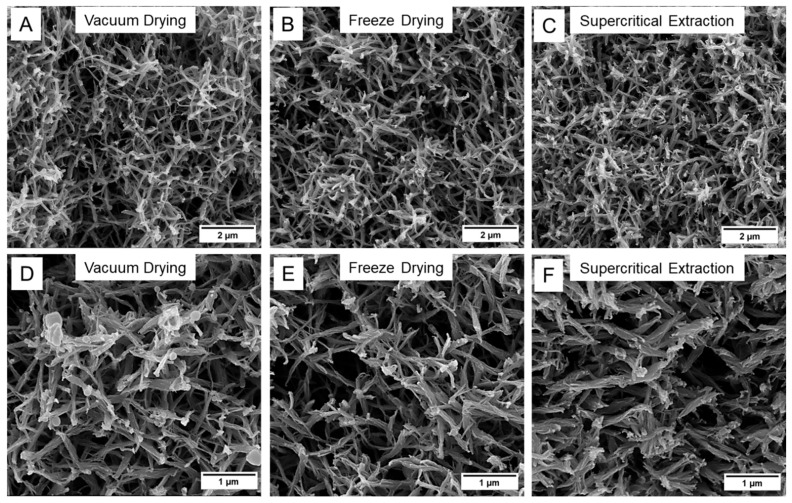
SEM micrographs of the PPS aerogels gelled from a 12.5 vol% PPS/DPA solution and dried with (**A**,**D**) vacuum drying, (**B**,**E**) freeze-drying, or (**C**,**F**) supercritical extraction. Images were collected at (**A**–**C**) 10 k× magnification or (**D**–**F**) 20 k× magnification.

SEM images collected on the PPS aerogel prepared from a 12.5 vol% PPS/DPA solution and dried with vacuum drying, freeze-drying, or supercritical extraction can be found in Figure 4. For the PPS aerogels prepared from an 8.3 vol% or a 16.8 vol% PEEK/DPA solution and dried with vacuum drying, freeze-drying, or supercritical extraction, SEM images detailing internal aerogel morphology can be found in Figures S6 and S7, respectively. Similar to the PEEK/DPA aerogels, all the PPS aerogels gelled in DPA displayed a strut-like morphology consisting of thin, fibrillar PPS axialites. The PPS aerogels displayed minimal differences in axialite size, surface texture, and pore size when compared across the different drying techniques, as observable by SEM. The drying technique appears to have little noticeable impact on the PPS network in strut-like PPS aerogels prepared from DPA solutions.

Previous work found that the more rigorous extraction methods of vacuum drying or freeze-drying can cause significant deformation, observable through SEM, when compared to supercritical drying [25,26,27]. Where open networks of fine pores are attained for gels dried with supercritical drying, large macropores and compressed fine porosity are commonly observed in gels dried with freeze-drying [25,26,27,29,30]. Xerogels prepared with evaporative drying techniques tend to have a considerably denser appearance observed in SEM, where much of the porosity has disappeared [25,26,27,31,32]. Any differences observed in the SEM images on PEEK or PPS aerogels dried using different techniques are considerably more subtle than the differences commonly observed between drying methods for other polymer aerogels.

**Figure 5:**
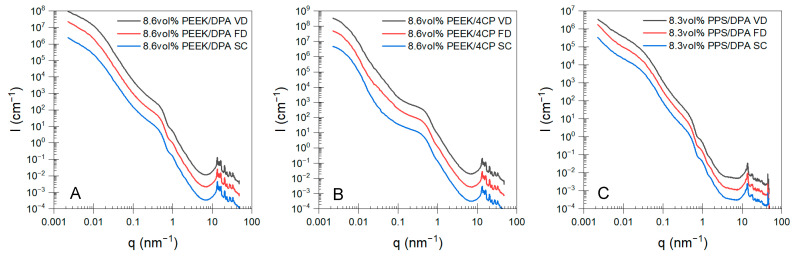
Merged USAXS/SAXS/WAXS profiles for the aerogels prepared from (**A**) 8.6 vol% PEEK/DPA, (**B**) 8.6 vol% PEEK/4CP, and (**C**) 8.3 vol% PPS/DPA solutions. Profiles are vertically shifted for clarity.

X-ray scattering experiments were performed on the PEEK and PPS aerogels, cryogels, and xerogels prepared with different drying techniques in order to determine the effect of the drying method on the hierarchical aerogel structure over a wide range of length scales. Merged USAXS/SAXS/WAXS profiles for the aerogels prepared from solutions of 8.6 vol% PEEK/DPA, 8.6 vol% PEEK/4CP, or 8.3 vol% PPS/DPA can be found in Figure 5A, Figure 5B, and Figure 5C, respectively. Merged USAXS/SAXS/WAXS profiles for the aerogels prepared at different concentrations can be found in Figure S8. The hierarchical morphology of these semicrystalline polymer aerogels, consisting of numerous structural levels, was previously described in detail in our previous publications [11,13].

Over the broad q-range probed here, it is clear that the PEEK and PPS aerogels display similar hierarchical features, consistent with the similar morphologies as observed by SEM. Briefly, a broad knee is observed from 0.01–0.03 nm^−1^, which is assigned to a dimension associated with the PEEK or PPS lamellar aggregates (i.e., tentatively the axialite thickness). For aerogels formed from PEEK/4CP solutions, this feature is likely associated with the diameter of the spherical aggregates. At higher *q*, a second knee is observed in all aerogel systems between 0.2 nm^−1^ and 0.8 nm^−1^, which is associated with the thickness of polymer crystalline lamellae within the axialites. Within the WAXS region, (i.e., *q* above 7 nm^−1^), numerous sharp diffraction peaks are observed, which are assigned to the crystalline reflections of the orthorhombic unit cell characteristic of PEEK [33,34] or the orthorhombic unit cell characteristic of PPS [35,36]. An in-depth analysis of scattering profiles utilizing the unified equation [37] to extract the size and fractality of the hierarchical aerogel structure can be found in the supporting information. With the strut-like morphologies, the drying method was not found to significantly affect the morphology of the aerogels prepared from PEEK or PPS solutions in DPA. However, for the globular aerogels prepared from PEEK/4CP solutions, the radius of gyration (Table S1) of both the crystallite feature and the aggregate feature was found to be smaller for the systems prepared with vacuum drying or freeze-drying compared to the systems prepared with supercritical extraction. The surface roughness of the aggregate feature was found to be higher for the PEEK/4CP aerogels prepared with vacuum drying or freeze-drying, compared to the samples prepared with supercritical drying. In general, the drying method appears to have no significant effect on the strut-like network morphology of PEEK or PPS aerogels prepared from DPA as probed by SAXS and SEM. However, some deformation of both levels of the network structure of the globular PEEK aerogels prepared from 4CP was observed with X-ray scattering analysis, which may have an impact on the macroscopic properties of these aerogels.

### 2.2. Aerogel Shrinkage 

**Figure 6:**
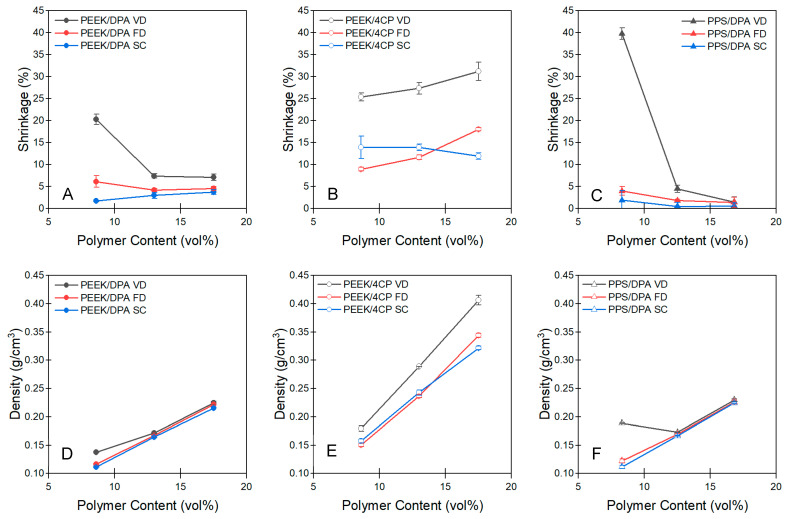
Shrinkage of the aerogels prepared from (**A**) PEEK/DPA solutions, (**B**) PEEK/4CP solutions, and (**C**) PPS/DPA solutions. Density of the aerogels prepared from (**D**) PEEK/DPA solutions, (**E**) PEEK/4CP solutions, and (**F**) PPS/DPA solutions.

Our previous research [11] indicated that network morphology likely has a significant impact on how an aerogel responds to the forces of drying. Macroscopic shrinkage would be expected in the case of a gel structure being weak relative to the forces induced by vacuum drying or freeze-drying. Volumetric shrinkage of the PEEK and PPS aerogels prepared from DPA or 4CP solutions and dried using different drying techniques can be found in Figure 6A–C. Tabulated aerogel properties, including shrinkage, can be found in Table S2. Images of the PEEK aerogels prepared from DPA or 4CP solutions in the wet state and dried using vacuum drying, freeze-drying, or supercritical drying can be found in Figure S9.

For the aerogels prepared from PEEK/DPA solutions (Figure 6A), shrinkage was found to be highest for vacuum-dried xerogels, then freeze-dried cryogels, then supercritically extracted aerogels. Vacuum drying causes the most gel shrinkage, as capillary forces can be large [17]. Freeze-drying is considerably gentler than vacuum drying on gel morphology, but ice freezing can cause some internal network deformation and shrinkage [21]. Finally, supercritical extraction is the gentlest extraction method, as going around the supercritical point avoids the liquid–vapor meniscus responsible for capillary forces [38]. Shrinkage values for the PEEK/DPA aerogels were found to be low, with values below 8% for all the gels except for the 8.6 vol% PEEK/DPA gel sample that was vacuum-dried. It is well-known that gels prepared at low polymer concentrations have a lower modulus [11], and as such it is believed that this low gel concentration results in a gel that is too weak to resist the capillary forces and therefore shrinks considerably. With increasing PEEK content in a PEEK/DPA solution, gels dried using vacuum drying and freeze-drying tended to shrink less. It is believed these denser gels resist the forces of shrinkage better than gels prepared at lower PEEK concentrations. In general, the globular PEEK aerogels made from solutions in 4CP (Figure 6B) showed the most shrinkage across all the drying methods. In contrast, the strut-like aerogels with PEEK (Figure 6A) and PPS (Figure 6) were much more resistant to shrinkage across all the drying methods. Moreover, the strut-like aerogels prepared through supercritical extraction exhibited the least shrinkage, but only slightly less than the freeze-dried aerogels.

The strut-like morphology of gels prepared from DPA is believed to resist the stresses of drying better than the weaker globular morphology of gels prepared from 4CP. For the PEEK gels prepared from 4CP solutions, shrinkage was found to be highest for xerogel samples that were vacuum-dried. Freeze-drying and supercritical drying were found to have comparable shrinkage, on average. Considerable shrinkage caused by freeze-drying in cryogel samples is not unexpected due to the delicate internal morphology of PEEK/4CP aerogels. Considerable shrinkage, above 10%, was observed in all the PEEK/4CP samples, even those prepared with supercritical extraction. Despite being regarded as the most gentle drying method, supercritical drying can still result in measurable shrinkage, with condensation of solvent vapors within the gel structure after depressurization being a possible cause [22]. Capillary forces induced by the evaporation of residual ethanol remaining after incomplete supercritical extraction are believed to cause the shrinkage observed in samples prepared with supercritical extraction.

In the case of the PPS aerogels prepared from DPA solutions (Figure 6C), shrinkage was found to be highest for the vacuum-dried samples, followed by the freeze-dried samples, then the supercritically extracted samples. The shrinkage of PPS aerogels tends to decrease with increasing polymer content, which is similar to the behavior of PEEK aerogels prepared from DPA. With the exception of the 8.3 vol% PPS aerogel, shrinkage of the PPS aerogels was slightly lower than the shrinkage of the PEEK aerogels prepared from DPA (Figure S10a). The forces exerted on the gel network during drying are similar to the previously described, with capillary forces induced by vacuum drying causing the largest shrinkage, followed by ice crystal growth on freezing inducing a lower amount of shrinkage, then supercritical extraction avoiding excessive stresses through its extraction route. Shrinkage values for the PPS/DPA aerogels were observed to be low, with values below 5% for all the gels, except for the 8.3 vol% PPS/DPA gel sample that was vacuum-dried. As this gel was prepared at a low polymer concentration, its lower modulus (see below) rendered this aerogel more susceptible to network deformation during drying.

As liquid is removed from the gel structure, the gels may densify due to the relationship between gel strength and the forces exerted during drying. The density of the aerogels derived from solutions of PEEK/DPA, PEEK/4CP, or PPS/DPA can be found in Figure 6D, Figure 6E, and Figure 6F, respectively. Tabulated values for density can be found in Table S2. As expected, for each series, density increased with increasing polymer content in the solution prior to gelation. Increasing the polymer content increases the amount of solid polymer within the gel volume, thereby increasing the density of the resulting aerogel. With respect to the drying method, vacuum drying tends to result in the highest density, followed by freeze-drying and supercritical drying, which are generally comparable. The differences between the densities of the gels prepared using different drying methods were most obvious for the strut-like aerogels prepared from 8.6 vol% PEEK/DPA or 8.3 vol% PPS/DPA. Differences in density were minimal for the gels prepared at higher polymer content with changing drying method for the strut-like aerogels. For the globular aerogels prepared from PEEK/4CP solutions, the aerogel densities were all higher than those of the strut-like aerogels. For these globular aerogels, vacuum drying tended to result in aerogels with a higher density than the aerogels dried with freeze-drying or supercritical extraction across all the polymer contents investigated.

As gel modulus is largely responsible for resisting the forces exerted during any drying method [17,21,24], it is believed that weaker gels, for example, globular aerogels, or strut-like aerogels prepared from relatively dilute 8.6 vol% PEEK/DPA solutions, experience a larger shrinkage than more robust gels. It is also important to consider the driving forces that cause shrinkage. In the case of vacuum drying, the pore size of gels is an important factor to consider, as capillary pressure is related to the inverse of pore radius (Equation (1)). Next, we will characterize the pore structure of our aerogels, as well as the mechanical strength of the gels. Finally, we will verify our results through the application of a shrinkage model that considers both pore size and gel strength.

### 2.3. Nitrogen Porosimetry of Aerogels 

**Figure 7:**
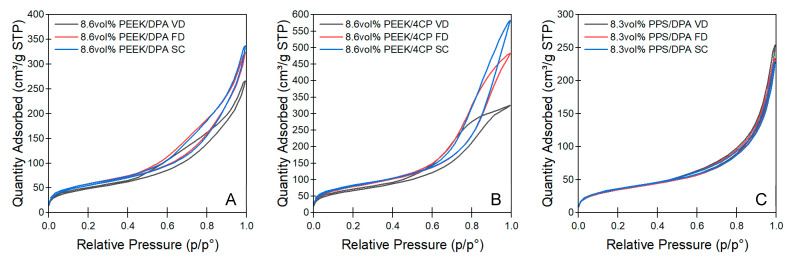
Nitrogen sorption isotherms of the aerogels prepared from (**A**) 8.6 vol% PEEK/DPA, (**B**) 8.6 vol% PEEK/4CP, and (**C**) 8.3 vol% PPS/DPA solutions.

Nitrogen adsorption experiments are useful to investigate the microscopic pore geometries of aerogels. Nitrogen porosimetry yields information on the surface area of porous adsorbents, as well as their mesoporous character. Nitrogen adsorption isotherms for the aerogels prepared from 8.6 vol% PEEK/DPA, 8.6 vol% PEEK/4CP, or 8.3 vol% PPS/DPA solutions can be found in Figure 7A, Figure 7B, and Figure 7C, respectively. Nitrogen sorption isotherms for the aerogels prepared at different solution concentrations can be found in Figure S13. Nitrogen isotherms are classified by their shape, which is characteristic of a specific type of adsorbent. Nitrogen isotherms collected on the aerogels in this study all displayed an inflection point at a low relative pressure (below p/p^o^ = 0.05), which is associated with the transition between monolayer and multilayer adsorption, and a hysteresis loop above the relative pressures of p/p^o^ = 0.4, associated with pore condensation [39]. These features are all commonly attributed to an IUPAC type IV nitrogen isotherm, which is characteristic of a mesoporous adsorbent with 2–50 nm pores [39]. For all the aerogels, the hysteresis loop displaying unlimited adsorption approaching relative pressures of p/p^o^ = 1.0 was characterized as IUPAC type H3 hysteresis, which is characteristic of slit-like mesopores formed from aggregates of plate-like particles [39].

The shape of the hysteresis loop varied among the different aerogel systems. For the PEEK/DPA aerogels, the sickle-shaped hysteresis loop observed was wider closer to p/p^o^ = 0.4 than p/p^o^ = 1.0. This shape of the hysteresis loop has been observed in adsorbents with a bimodal distribution of pores where pore blocking may be relevant [40]. The PEEK aerogels prepared from 4CP solutions displayed a broad hysteresis loop with a wedge shape for the samples dried with supercritical extraction. The aerogels dried with vacuum drying or freeze-drying have a more sigmoidal shape, with a notable decrease in quantity, adsorbed at high relative pressure values above p/p^o^ = 0.8 compared to similar samples dried with supercritical extraction. Additionally, the supercritically extracted aerogels prepared from 4CP tended to have a constant slope at high relative pressure values toward p/p^o^ = 1.0, unlike the PEEK aerogels prepared from DPA, which had an increasing slope at high relative pressure values. Furthermore, the vacuum-dried and freeze-dried aerogels prepared from 4CP tended to have decreasing slopes approaching high relative pressure values, as they appeared to be approaching a limiting adsorption value. A limiting adsorption value and nearly parallel adsorption and desorption profiles over the hysteresis loop are the defining features of a type H1 hysteresis loop, which is characteristic of mesoporous adsorbents with a narrow distribution of pore sizes [39]. The hysteresis loops for the PEEK aerogels gelled in 4CP can be described as a hybrid between type H1 and H3 hysteresis. The gels prepared from 4CP and dried by vacuum drying displayed considerably more H1 character, while the aerogels dried through supercritical extraction were more comparable to the H3 hysteresis loops, and the freeze-dried aerogels displayed hysteresis behavior between the two other drying methods. The fact that the hysteresis loop displayed hybrid behavior moving from being more similar to H3 hysteresis to being more similar to H1 hysteresis moving from supercritically dried aerogels to vacuum-dried aerogels may indicate that the pore size distribution was moving to smaller pore sizes, as the closure point became more visible at high relative pressures, approaching p/p^o^ = 1.0, for the vacuum-dried samples. While the nitrogen isotherms collected on PPS aerogels generally appear similar in shape to those collected on PEEK/DPA aerogels, the hysteresis loop observed for the PPS aerogels was considerably narrower than that observed for the PEEK/DPA aerogels. Additionally, the hysteresis loop displayed the same width across the whole loop for the PPS aerogels, whereas the hysteresis loop for the PEEK/DPA aerogels was considerably wider at the loop closure point, approaching relative pressures of 0.4.

**Figure 8:**
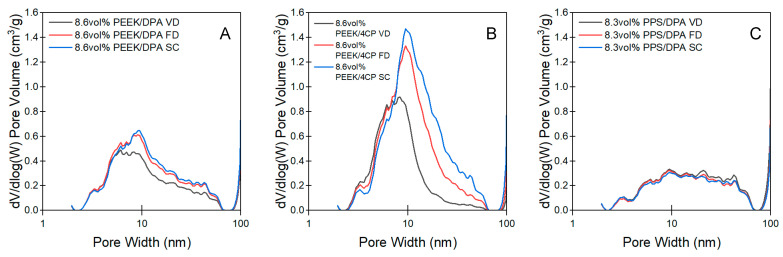
Pore size distribution of the aerogels prepared from (**A**) 8.6 vol% PEEK/DPA, (**B**) 8.6 vol% PEEK/4CP, and (**C**) 8.3 vol% PPS/DPA solutions.

Non-local density functional theory (NLDFT) is a powerful analysis technique that was used to extract pore size distributions from nitrogen isotherms through modeling the isotherms with a theoretical adsorbent system [41]. Figure 8A–C shows representative pore size distributions for the aerogels prepared from 8.6 vol% PEEK/DPA, 8.6 vol% PEEK/4CP, or 8.3 vol% PPS/DPA solutions. Pore size distributions for the aerogels prepared at higher polymer concentrations can be found in Figure S14. All pore size distributions for the PEEK aerogels (Figure 8A and Figure S14a,b) prepared from DPA displayed a peak at a pore width of about 8–10 nm and had a considerable shoulder, extending to ca. 60 nm. Pore size distributions were analyzed to yield the full peak width at half maximum (FWHM) (Figure S15). No significant trends with the drying method were observed in the FWHM analysis, other than all the distributions being fairly broad. In addition, pore volume across pore sizes of 6–60 nm tended to decrease the most for the vacuum-dried samples, compared to the samples prepared using supercritical drying, with the 8.6 vol% PEEK/DPA series suffering from the largest decrease in pore volume across this region. As shrinkage was highest for the 8.6 vol% series and for the vacuum-dried xerogels, it can be inferred that some of this shrinkage diminished the mesopore volume for these samples.

The pore size distributions obtained by applying NLDFT to nitrogen sorption isotherms collected on the PEEK aerogels prepared from 8.6 vol% PEEK/4CP solutions can be found in Figure 8B and Figure S14c,d. Each pore size distribution collected from the aerogels prepared from PEEK/4CP solutions displayed a single monomodal peak. For the samples dried using supercritical extraction or freeze-drying, the peak was located at a pore width of about 10 nm, while the vacuum-dried samples had the peak pore size located at about 8 nm. Peak broadness, assessed with the FWHM (Figure S15), was found to be lowest for the vacuum-dried samples, higher for the freeze-dried samples, and the highest for the samples dried with supercritical extraction. Increasing the aggressiveness of the drying method, from supercritical extraction, to freeze-drying, to vacuum drying, tended to narrow the pore size distribution and move the mean pore size to smaller sizes. This trend displays the effect of shrinkage on mesopores found in the PEEK gels prepared from 4CP: with increasing shrinkage (as a result of increasing the aggressiveness of the drying method), the pore size distribution moves to smaller pore sizes. Compared to the PEEK aerogels prepared from DPA, the PEEK aerogels prepared from 4CP had a narrower, monomodal distribution, whereas the gels prepared from DPA had a broad, bimodal distribution. Pore shrinkage of the PEEK/4CP aerogels was attributed to the increase in power law scattering observed in X-ray scattering profiles. The surfaces of the PEEK network were most susceptible to damage from drying methods, which resulted in rougher surfaces, thereby increasing the power law scattering observable in X-ray scattering (Figure 5B and Figure S8c–d).

Pore size distributions for the PPS aerogels can be found in Figure 8C and Figure S14e,f. The pore size distributions for the PPS aerogels were very broad and monomodal, with a peak pore size centered around 20 nm. The PPS aerogels did not display significant differences in their pore size distributions with the drying method or with the PPS content. The average pore size for the PPS aerogels was considerably larger than that of the PEEK aerogels. Compared to the PEEK aerogels, the total pore volume probed by nitrogen was lower for the PPS aerogels. The broadness of the pore size distribution, as assessed by the FWHM (Figure S15), was considerably larger for the PPS aerogels than for the PEEK aerogels.

NLDFT analysis also yields the specific mesopore volume. This allows evaluation of the total porosity by pore size. An in-depth analysis of pore volume can be found in the supporting information. It was found that the aerogels prepared from PEEK/4CP solutions tended to have a considerable proportion of their open porosity consisting of mesopores (with pore sizes between 2 nm and 50 nm), up to 35%, compared to 5–15% for the PEEK and PPS aerogels prepared from DPA. The aerogels prepared from PEEK/4CP solutions appeared to be more susceptible to capillary forces due to the prominence of these fine pores.

**Figure 9:**
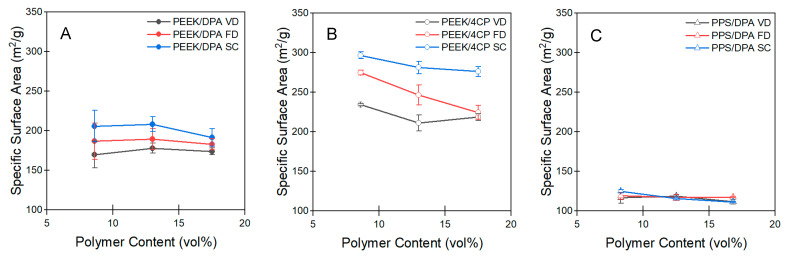
Specific surface area of the aerogels prepared from (**A**) PEEK/DPA solutions, (**B**) PEEK/4CP solutions, and (**C**) PPS/DPA solutions.

Specific surface area can also be determined with nitrogen porosimetry through the application of the Brunauer–Emmett–Teller (BET) theory [42]. Specific surface areas determined by the BET theory can be found in Figure 9A–C. Table S2 displays the tabulated values for the aerogel surface area. For all the PEEK aerogels, surface area was lowest for the aerogels prepared by vacuum drying, freeze-drying featured higher surface areas, and the aerogels prepared by means of supercritical drying tended to have the highest surface areas. Supercritical drying is the gentlest drying method, so it preserves fine porosity the best out of the drying methods, leading to the highest surface areas. The freezing of ice causes compression on the gel matrix, which can lead to disruption of the fine surfaces of the gel structure, decreasing surface area. Vacuum drying exerts the most force on the gel matrix through the fine pores, which is destructive to surface area. Surface area tends to slightly decrease with increasing PEEK content. Increasing the volume occupied with PEEK also increases the probability that PEEK aggregates will overlap. The aerogels prepared from DPA tended to have considerably lower surface areas (ranging from 170 to 205 m^2^/g) than the aerogels prepared from 4CP (ranging from 210 to 295 m^2^/g). The finer spherical aggregates (Figure 3) produced during gelation in 4CP may have more surface area accessible than the larger strut-like aggregates formed during gelation in DPA (Figure 2). The PPS aerogels prepared from DPA showed lower surface areas, ca. 120 m^2^/g. No significant difference in the polymer content or the drying method on surface area was found for the PPS aerogels. As the mesopore volume was lowest in the PPS aerogels (Figure S17a–c), this may have limited the amount of available surface area for gas adsorption. The surfaces of the features of the PPS aerogels as observed by SEM (Figure 4) did not appear to be as textured as those of the PEEK aerogels, which may indicate a lower area available for gas adsorption.

### 2.4. Mechanical Properties of Aerogels 

**Figure 10:**
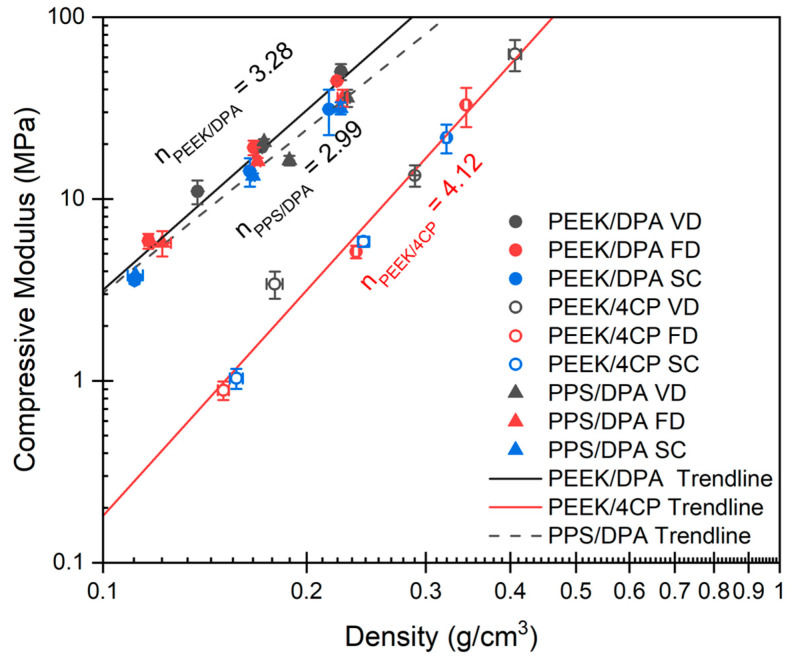
Compressive modulus vs. aerogel density for the polymer aerogels. Coefficient of determination for the trendline fits are R^2^ = 0.9498 for the PEEK/DPA aerogels, R^2^ = 0.9594 for the PPS/DPA aerogels, and R^2^ = 0.9958 for the PEEK aerogels.

As previously stated, it is believed that the modulus of an aerogel largely determines its behavior under the stresses that occur during drying, which, if greater than the maximum stress that can be supported by the gel structure, causes shrinkage. As aerogel modulus scales exponentially with bulk density, the compressive modulus of the PEEK aerogels is presented on a logarithmic scale versus the aerogel bulk density, as shown in Figure 10.

Mechanical properties stemming from the gel morphology are crucial to resisting deformation caused by forces induced during drying. Porous materials display a power law relationship between bulk density and modulus [43]. With increasing density, more solid material is available to distribute the stresses exerted on the material. The exponent used to describe the power law scaling between modulus and density indicates the efficiency of the network in distributing stresses [44]. The ability of the porous material to distribute stresses effectively is related to the connectivity of the network [43]. For example, connectivity of an aerogel can change considerably with density, and aerogels tend to have scaling exponents between 3 and 4 [44]. Poorly connected systems, such as a globular particulate networks, ineffectively distribute stress among the network features, leading to a higher exponent. Networks with high connectivity make efficient use of the network material, evidenced by a lower exponent. Cellular networks, the most efficient isotropic material, have an exponent of 2.0 [43].

The samples prepared with vacuum drying (xerogels) tended to have higher densities and compressive moduli than the samples prepared with freeze-drying or supercritical extraction. Vacuum drying shrinks the gel more, meaning it increases the density of the gel, which means a larger fraction of the volume of the aerogel is taken up by the solid network, thereby increasing the gel’s modulus. Compressive modulus tends to increase with increasing polymer volume fraction, as modulus increases with increasing aerogel density [43]. Stress–strain curves for the PEEK and PPS aerogels can be found in Figure S21. The strut-like aerogels prepared from PEEK/DPA (Figure S21a–c) or PPS/DPA (Figure S21g–i) solutions tended to display ductile compaction, with stress increasing smoothly with increasing strain, while the globular aerogels prepared from PEEK/4CP (Figure S21d–f) tended to show brittle fracture, as indicated by sharp decreases in stress after yield.

The aerogels prepared from PEEK/DPA followed a power law relationship with density and the aerogels prepared from PEEK/4CP also followed their own power law scaling relationship with density. At comparable densities, the aerogels prepared from PEEK/DPA solutions displayed a higher compressive modulus than the aerogels prepared from PEEK/4CP solutions. The scaling exponent *n* was found to be 3.28 ± 0.23 for the aerogels prepared from PEEK/DPA solutions and 4.12 ± 0.24 for the aerogels prepared from PEEK/4CP solutions. This means, in agreement with our previous work [11], that strut-like aerogels more efficiently distribute compressive stresses than globular aerogels. Furthermore, the aerogels prepared from PPS/DPA solutions displayed similar modulus/density scaling as the aerogels prepared from PEEK/DPA solutions. Both strut-like systems behaved nearly identically under compression. The scaling exponent *n* for the PPS aerogels was found to be 2.99 ± 0.20, which is comparable to the 3.28 found for the PEEK aerogels prepared from DPA. The compressive modulus of the aerogels studied in this work is solely based on network connectivity and bulk density. Interestingly, the drying method does not impact the modulus outside of dictating the bulk density through shrinkage. Yield stress was also found to display power law relationships with density, as observed in Figure S22.

**Figure 11:**
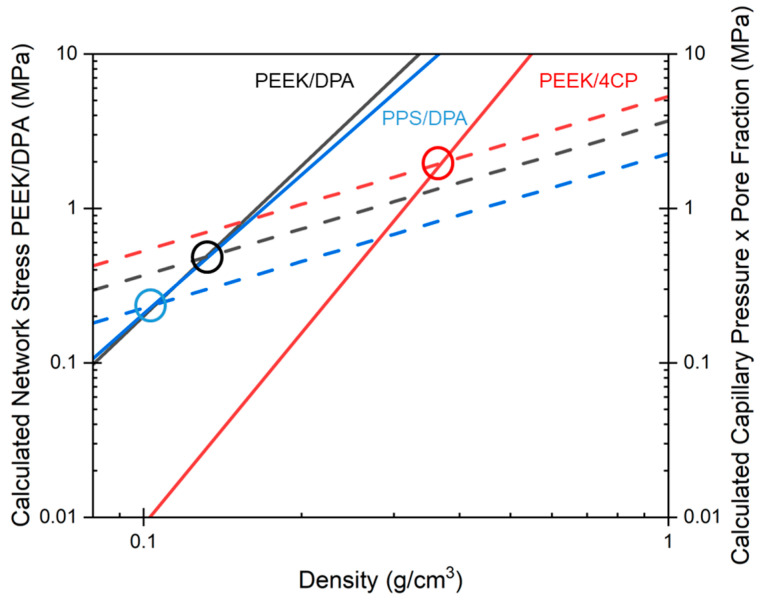
Calculated network stress (shown as solid lines) and calculated capillary pressure (shown as dashed lines) as a function of density for the aerogels prepared from 8.6 vol% PEEK/DPA (shown in black), 8.6 vol% PEEK/4CP (shown in red), and 8.3 vol% PPS/DPA (shown in blue) solutions.

Characterization of the mechanical behavior of PEEK aerogels allows for the modeling of aerogel shrinkage. Knowing how the modulus scales with density allows an estimate of the maximum network stress the gel structure is able to resist, and knowing the pore structure allows the estimation of the maximum forces exerted on a gel by capillary forces during vacuum drying [17]. Figure 11 shows the calculated network stress and capillary pressure plotted against gel density for the aerogel systems at 8.6 vol% PEEK or 8.3 vol% PPS. Network stress is calculated using the modulus–density scaling exponent *n*, the minimum density, *ρ_min_*, and the estimated modulus at this minimum density. The stress induced by capillary pressure is calculated using aerogel surface area (assuming that aerogels dried under supercritical conditions have surface area comparable to the gels prior to drying), the surface tension of ethanol at 60 °C, and the contact angle of ethanol on PEEK or PPS. The calculated network stress and capillary pressure plots for each aerogel system at the different polymer concentrations can be found in Figure S23. Increasing the polymer concentration decreases the surface area of the resulting aerogel, thereby slightly decreasing the capillary pressure. The differences in the capillary pressure curves due to polymer concentration are small and likely do not have a significant effect.

For each aerogel system, the plots in Figure 11 take the form of two intersecting lines; the capillary pressure line has a low slope and the network stress line has a high slope. This indicates that for the gels prepared at low densities, stresses induced by capillary pressure were greater than the maximum stress the network was able to support, and thus, the gel shrunk until a gel density was reached where the maximum network stress was greater than the stresses induced by capillary forces. In addition, gels prepared at densities higher than the intersection of network stress and capillary pressure should be sufficiently strong to resist the forces exerted by capillary pressure, and thus shrinkage should be minimal.

For the gels prepared from PEEK/DPA, the network stress line intersected the capillary pressure line at a density of about 0.14 g/cm^3^, while for the gels prepared from PEEK/4CP, this intersection occurred at 0.37 g/cm^3^. Due to their superior compressive properties, the gels prepared from PEEK/DPA could be made at considerably lower densities without experiencing significant shrinkage, when compared to the gels prepared from PEEK/4CP. Comparing the calculated network stress curves, it is apparent that PEEK/DPA gel systems are able to support considerably more stresses than PEEK/4CP gel systems at the same densities. While the gels prepared from PEEK/4CP displayed higher surface areas than the gels prepared from PEEK/DPA, the shift in the capillary force curve was small between the two systems. For PEEK systems, this indicates that the gel modulus, dictated by the gel morphology, is the most important factor in determining shrinkage.

For the PPS gels prepared from DPA solutions, the network stress line intersected the capillary pressure line at a density of about 0.11 g/cm^3^, while the gels prepared from PEEK/DPA solutions had this intersection occurring at 0.14 g/cm^3^. As the PPS aerogels had a lower surface area than the PEEK aerogels prepared from DPA, the capillary pressure exerted on them was lower. Due to the lower pressure exerted on the PPS aerogels during vacuum drying, the PPS aerogels displayed a lower shrinkage than the PEEK aerogels prepared from DPA. As the compressive modulus of PPS aerogels and PEEK aerogels prepared from DPA solutions displays a similar scaling relationship, the difference in capillary pressure exerted during drying is the largest factor determining the difference in shrinkage between PPS aerogels and PEEK aerogels prepared from DPA.

The results of the network stress modeling are in good agreement with the experimentally observed shrinkage. For the gels prepared from PEEK/DPA solutions, the 8.6 vol% PEEK concentration resulted in gels at low densities where the stress exerted by capillary forces were greater than the strength of the network. Shrinkage occurred until the network became stiff enough to resist capillary forces. For the gels prepared from PEEK/DPA solutions at higher PEEK concentrations, the gels had sufficient modulus to resist capillary forces, and thus these gels shrunk minimally. For the gels prepared from PEEK/4CP solutions, each concentration was below the point where capillary forces exceeded network strength. Considerable shrinkage occurred at each concentration. At the comparable polymer content, the PPS aerogels shrunk less than the aerogels prepared from PEEK/DPA solutions. As the compressive modulus of the PPS and PEEK/DPA aerogels was similar, and the capillary forces exerted on the PPS aerogels were lower than the capillary forces exerted on the PEEK aerogels prepared from DPA, the PPS aerogels better resisted capillary forces. Due to the lower capillary pressure, the PPS aerogels shrunk less than the PEEK aerogels prepared from DPA.

The aerogels prepared using low polymer contents, 8.3 vol% PPS/DPA or 8.6 vol% PEEK/DPA, shrunk considerably more than the gels prepared at higher polymer concentrations due to their lower compressive modulus. The PPS gels shrunk considerably more than the PEEK gels. The power law relationship for PEEK/DPA systems underpredicted the modulus of the vacuum-dried xerogel samples (Figure 10), while the power law relationship for PPS/DPA systems overpredicted the modulus of the samples prepared with vacuum drying. Thus, the PEEK aerogels prepared from DPA deviated from the calculated behavior with lower shrinkage, while the PPS aerogels prepared from DPA deviated from the calculated behavior with a higher shrinkage.

Therefore, gel modulus is critical for resisting capillary forces on vacuum drying. Moreover, modulus is important for freeze-dried gels as it resists the growth of internal ice crystals. Gel modulus is even important for gels prepared using supercritical extraction, as they also experience forces that lead to shrinkage, despite being subjected to the gentlest extraction method.

## 3. Conclusions

In this work, we prepared monolithic semicrystalline polymeric aerogels using vacuum drying, freeze-drying, and supercritical drying. We found that the drying method directly influenced the shrinkage and final density of gels, as well as determined the fine mesopore texture of gels. The PEEK aerogels displayed more shrinkage than the PPS aerogels. The PEEK aerogels that were prepared using the more aggressive drying methods of vacuum drying or freeze-drying were found to produce aerogels with smaller mesopores (where mesopores are defined as pores with pore widths between 2 and 50 nm), lower mesopore volume, and lower surface area than the aerogels prepared using the gentler supercritical extraction method. The globular PEEK aerogels prepared from 4CP tended to be more sensitive to the drying method and were prone to larger changes in shrinkage, density, mesopore size, mesopore volume, and surface area than the strut-like PEEK aerogels derived from DPA. The PPS aerogels displayed a considerably lower sensitivity to the drying method, with no noticeable differences observed for the mesopore volume. The modulus of aerogels was found to be dependent on the connectivity of the aerogel network and density of the dry gel, and the drying method had no direct impact on gels’ mechanical properties. The robust PEEK or PPS aerogels prepared with fibrillar morphologies were found to have superior mechanical properties to the globular PEEK aerogels and were thus able to resist the forces exerted during drying. The PPS aerogels were found to have relatively large mesopores compared to the PEEK aerogels prepared from DPA, experiencing lower capillary stresses and, thus, shrinking less. The calculated maximum network stress that could be supported by the aerogel was compared to the capillary pressure induced on the aerogel through vacuum drying, and the comparison of these values was in good agreement with the experimental results. This work emphasizes that network connectivity plays a critical role in resisting collapse during the process of gel drying. Furthermore, carefully tailoring gel properties, such as modulus and pore size, can allow aerogel processing with the simpler evaporative drying method. Future work will involve expanding our methods of preparing aerogel networks with increased connectivity to better prepare aerogels from weaker matrix materials.

## 4. Materials and Methods

### 4.1. Materials

PEEK (Victrex 150P) and PPS (Ryton QA200N) were provided by Solvay Specialty Polymers (Alpharetta, GA, USA); 1,3-diphenylacetone (DPA) was purchased from Oakwood Chemical (Estill, SC, USA) or Sigma-Aldrich (St. Louis, MO, USA); 4-chlorophenol was purchased from ThermoFisher Scientific (Waltham, MA, USA); ethanol (200 proof, 100% USP, Decon Labs) was purchased from Fisher Scientific Company LLC (Suwanee, GA, USA); water-based conductive graphene carbon paint was purchased from Electron Microscopy Sciences (Hatfield, PA, USA). All polymers and chemicals were used as received.

### 4.2. Gel Preparation

Preparation of PEEK gels in DPA was similar to the protocols previously performed by our group [11]. PEEK and DPA were added to a three-neck round bottom flask equipped with an overhead stirrer, a condenser, and an argon inlet. Argon was chosen to provide an inert atmosphere for dissolution in order to prevent degradation of the polymer and solvent at elevated dissolution temperatures. Argon was allowed to purge the flask for at least 20 min. The flask was placed in a metal bath, using CS Alloys (Gastonia, NC, USA) Tru 281, bismuth–tin alloy (T_m_ = 138 °C), set at 320 °C. The flask was purged with argon during dissolution. Dissolution of PEEK in DPA occurred over 1 and 3 h, depending on the PEEK concentration. The solution was stirred for the final 20 min of dissolution. The hot PEEK solution was poured into open-ended cylindrical glass tubes, which were held in a Hart Scientific (Everett, WA, USA) 9122 well heater (with a well diameter of ~20 mm) set at 50 °C. The tubes had a nominal inner diameter of 9 mm and a length of 100 mm. To prevent solvent escaping the tubes, the bottom end of each tube was sealed with a rubber septum. Gelation of the solution occurred within 5 min. Twenty minutes after the solution was poured into the tubes, the PEEK/DPA gels, still within the tubes, were immersed in an ethanol bath to exchange the DPA with ethanol. To prevent DPA crystallization, the ethanol bath was set to 50 °C. The ethanol was discarded and replaced with fresh ethanol after 24 h and the gels were pushed out of the tubes. After an additional 24 h in the ethanol bath, the gels were moved to a Soxhlet extractor to remove any residual DPA with ethanol. Soxhlet extraction was allowed to occur over 4 days. From there, ethanol-soaked gels were taken for vacuum drying or extraction with supercritical CO_2_. To prepare gels for freeze-drying, the ethanol-soaked gels were exchanged with deionized water for 5 days in a water bath. The water was discarded and refreshed with fresh deionized water daily.

Preparation of PEEK/4CP gels was adapted from our previous work [13]. PEEK and 4CP were loaded into a single-neck round bottom flask. The flask was placed into an oil bath filled with high-temperature silicone oil. The flask was sealed with a rubber septum, and argon gas was purged into the flask for at least 20 min. The flask was then submerged into the oil bath set to 215 °C. The tubes supplying argon were removed after 20 min. Dissolution of PEEK took place between 1 h and 4.5 h, depending on the PEEK concentration. The solution was held at this temperature for 20 min. Then, the bath was cooled to 175 °C, and held at this temperature for 5 min. The PEEK/4CP solution was pulled into polypropylene syringes with a needle. The syringes had a nominal inner diameter of 9 mm and a length of 40 mm. The bath temperature was dropped to 175 °C to prevent warpage of the polypropylene syringes. The needles were sealed with a piece of rubber septa to prevent leakage of the solution. Syringes containing the PEEK/4CP solution were placed in a water bath set to 50 °C. Gelation of the solution took place within 10 min, up to 4 days for the lowest-concentration gels. After gelation, the needles were removed from the syringes, and the syringes were placed into an ethanol bath set to 50 °C for 3 to 7 days, after which the end of the syringe was cut off with a razor blade and the gel was pushed from the syringe. It was found that the gels needed a lengthy extraction time to become solid enough to resist falling apart on removal from the syringes. Ethanol was exchanged once. Then, after 24 h of solvent exchange, the gels were placed in a Soxhlet extractor. Soxhlet extraction was allowed to occur over at least 6 days. From there, ethanol-soaked gels were taken for vacuum drying or extraction with supercritical CO_2_. To prepare gels for freeze-drying, the ethanol-soaked gels were exchanged with deionized water for 5 days in a water bath. The water was replaced with fresh deionized water daily.

Fabrication of PPS aerogels was similar to the protocols demonstrated previously by our group [14]. PPS and DPA were loaded at a given weight ratio in a large test tube (25 mm × 225 mm) and purged with argon gas for 25 min. The mixture was then placed in a Hart Scientific (Everett, WA, USA) 9123 well heater (well diameter ~27 mm) at 270 °C. The PPS polymer dissolved within 10–20 min depending on the concentration, followed by 5–10 min of vigorous stirring with a glass rod to create a homogeneous solution. PPS/DPA solutions were then removed from the well heater and poured into small test tubes (10 mm × 75 mm) to create cylindrical samples. Once cooled, the gel-containing test tubes were submerged in a room-temperature ethanol bath overnight to begin the solvent exchange from DPA. Next, gels were removed from the test tubes and placed back into the ethanol for five days, replacing the ethanol once per day, yielding ethanol-soaked gels. For the specimens dried via freeze-drying, ethanol-soaked gels were exchanged with deionized water for 5 days, where the water was refreshed once every 24 h, yielding hydrogels.

The initial solution concentration was expressed as vol% in order to easily compare the different gel systems. The literature values of amorphous density of PEEK [45], 1.26 g/cm^3^, and PPS [46], 1.32 g/cm^3^, were used to calculate the polymer volume. The densities of liquid DPA and 4CP were measured to be 1.07 g/cm^3^ and 1.22 g/cm^3^, respectively. The concentration of polymer solutions used to create gels can be found in Table 1.

### 4.3. Drying of Gels

Gels were cut to a length of about twice the diameter prior to drying. The water-soaked gels were frozen overnight at −18 °C and then lyophilized (LabConco Corporation, Kansas City, MO, USA) over 24 h to yield freeze-dried (FD) PEEK aerogels (commonly referred to as cryogels). To prepare vacuum-dried (VD) aerogels (commonly referred to as xerogels), ethanol-soaked gels were put into a vacuum oven (National Appliance Company, Uniondale, NY, USA) at room temperature or 60 °C for 24 h. To prepare aerogels through supercritical extraction (SC) with CO_2_, ethanol-soaked gels were placed into a Leica (Wetzlar, Germany) EM CPD300 critical point dryer. Then, the solvent exchange chamber was filled with ethanol and cooled to 15 °C. The chamber was pressurized to about 5 MPa with the addition of CO_2_ at a rate of about 25 mL/min. After 7 min, the CO_2_ flow was stopped and the chamber was allowed to equilibrate for 2 min. Then, the chamber volume was exchanged with CO_2_ at a rate of about 33 mL/min. In the exchange process, the volume of the chamber was replaced with supercritical CO_2_ about eight times. The chamber was heated to 41 °C at 1 °C/min and the pressure was increased to above 74 MPa. After reaching 41 °C and 74 MPa, the chamber was degassed at 0.3 MPa/min. Supercritical drying occurred over the course of about 5 h.

### 4.4. Characterization

The morphology of the PEEK aerogels was evaluated with scanning electron microscopy (SEM) using a LEO (Zeiss, Oberkochen, Germany) 1550 field-emission scanning electron microscopy (FE-SEM) with in-lens detection. The samples were freeze-fractured and mounted on a stub using conductive graphene carbon paint or conductive carbon tape. The paint was allowed to dry in a fume hood prior to sputter-coating. Mounted samples were sputter-coated with either a 5 nm thick layer of iridium with a Leica (Wetzlar, Germany) EM ACE600 sputter coater or with a 10 nm thick layer of Pt/Pd with a Cressington (Watford, UK) 208HR sputter coater.

Small-angle X-ray scattering (SAXS) experiments were performed using a Xenocs (Grenoble, France) Xeuss 3.0 SAXS/WAXS beamline, equipped with a GeniX 3D Cu HFVLF microfocus X-ray source with a wavelength of 0.154 nm (Cu K_α_). The sample-to-detector distance was 43 mm for wide-angle X-ray scattering (WAXS), 370 mm for mid-angle X-ray scattering (MAXS), 900 mm for SAXS, and 1800 mm for extra-small-angle X-ray scattering (ESAXS). To collect ultra-small-angle X-ray scattering (USAXS) data, a Bonse-Hart camera was used. The q-range was calibrated using a lanthanum hexaboride standard for WAXS and a silver behenate standard for ESAXS, SAXS, and MAXS. Two-dimensional scattering patterns were obtained using a Dectris (Baden-Daettwil, Switzerland) EIGER 4M detector. The exposure times were 4 h for USAXS, 4 h for ESAXS, 2 h for SAXS, 1 h for MAXS, and 1 h for WAXS. The scattering data were reduced and corrected for thickness, transmission, and background using the XSACT software version 2.10.3. Slit-smeared USAXS data were desmeared using XSACT, yielding pinhole-equivalent data. ESAXS, SAXS, MAXS, and WAXS data were output on an absolute scale using direct beam intensity. USAXS, ESAXS, SAXS, MAXS, and WAXS profiles were merged using the XSACT software. The scattering profiles were vertically shifted to facilitate comparison. Scattering plots are presented as scattering intensity, *I*(*q*), versus scattering vector *q*, where *q* = (4*π*/*λ*)*sin*(*θ*), *θ* is one half of the scattering angle, and *λ* is the X-ray wavelength. Merged USAXS/SAXS/WAXS scattering profiles were fit to the unified function [37] using Irena [47].

Gel volume was calculated using the volume of a right cylinder:

(2) $$V=\pi r^{2}l$$

where *r* is the cylinder radius and *l* is the cylinder length. Shrinkage was measured as follows:

(3) $$\frac{V_{wet}-V_{dry}}{V_{wet}}*100\%$$

where *V_wet_* is the volume of either the ethanol-soaked wet gel measured immediately before drying in the case of vacuum-dried and supercritically dried gels or of the water-soaked wet gel measured immediately before freezing, in the case of freeze-dried gels. *V_dry_* is the volume of the aerogel after drying. Aerogel bulk density, *ρ_b_*, was calculated as follows:

(4) $$\rho_{b}=\frac{m}{V_{dry}}$$

where *m* is the mass of a dried aerogel. Porosity of the PEEK aerogels, as determined by DSC, *Π_DSC_*, was calculated as follows:

(5) $$\Pi_{DSC}=\frac{\frac{1}{\rho_{b}}-\frac{1}{\rho_{s}}}{\frac{1}{\rho_{b}}}\times 100\%$$

where *ρ_b_* is the bulk density and *ρ_s_* is the skeletal density, calculated as follows:

(6) $$\rho_{s}=\frac{{\%X}_{c}\rho_{c}+(100-{\%X}_{c})\rho_{a}}{100}$$

where %*X_c_* is the crystallinity determined by DSC, *ρ_c_* is the crystalline density of PEEK (1.400 g/cm^3^), and *ρ_a_* is the amorphous density of PEEK (1.263 g/cm^3^) [45].

DSC experiments were performed on the PEEK aerogels using a TA Instruments (New Castle, DE, USA) Discovery DSC 2500. The aerogels were first flattened with a hammer to reduce artifacts due to settling. The PEEK aerogels were sealed in aluminum Tzero DSC pans. The samples were heated at 10 °C/min to 400 °C. Crystallinity (%*X_c_*) was determined as follows:

(7) $${\%X}_{c}=\frac{{\Delta H}_{m}}{{\Delta H}_{m}^{o}}\times 100$$

where ${\Delta H}_{m}$ is the integral of the experimental melting endotherm on first heating and ${\Delta H}_{m}^{o}$ is the melting enthalpy for PEEK, 130 J/g [45].

PPS aerogel porosity was measured via helium pycnometry using a Micromeritics (Norcross, GA, USA) AccuPyc 1340 helium pycnometer outfitted with a 3.5 cm^3^ insert and an equilibration rate of 0.03 psig/min. Porosity of the PPS aerogels, as determined by pycnometry, *Π_pyc_*, was calculated using the following equation:

(8) $$\Pi_{pyc}=\left[\frac{\left(V_{dry}-V_{spec}\right)}{V_{dry}}\right]\times 100\%$$

where *V_spec_* is the skeletal volume measured by the pycnometer.

Nitrogen porosimetry experiments were performed on aerogels using a Micromeritics (Norcross, GA, USA) 3 Flex gas adsorption analyzer. The PEEK aerogels were outgassed at 100 °C for 24 h. The PPS aerogels were degassed for 5 h at 100 °C. Adsorption and desorption isotherms were collected using nitrogen as the adsorbent at 77 K. Surface area was calculated using the Brunauer–Emmett–Teller (BET) method [42]. Pore size distributions and specific mesopore volume were obtained using nonlocal density functional theory (NLDFT), assuming slit-like pores. Pore size distributions were analyzed, yielding the full width at half maximum (FWHM). Specific pore volume was calculated as follows:

(9) $$V_{p}=\frac{1}{\rho_{b}}-\frac{1}{\rho_{s}}$$

Specific macropore volume was calculated by subtracting specific mesopore volume from specific pore volume. Mesopore content was calculated as follows:

(10) $$\frac{V_{meso}}{V_{p}}\times 100\%$$

where *V_meso_* is the specific mesopore volume.

Compression testing experiments were performed on aerogels using an Instron (Norwood, MA, USA) 3340 Universal Testing System with a 5 kN load cell or using an Instron 5944 Universal Testing System with a 2 kN load cell. Samples were tested in accordance with ASTM D695-23 [48]. Aerogel samples for compression testing were cylinders and complied with a 2:1 ratio of length to diameter. Offset yield strength was taken at the stress at which the stress–strain curve departed from linearity by 0.002 mm/mm strain. The modulus and density data were fit to a power law equation:

(11) $$\frac{E}{E_{solid}}\sim {\left(\frac{\rho }{\rho_{solid}}\right)}^{n}$$

where *E* is the modulus of the porous material, *E_solid_* is the modulus of the non-porous solid material (*E_solid,PEEK_* = 3600 MPa) [49], *ρ* is the density of the porous material, *ρ_solid_* is the density of the non-porous solid material (*ρ_solid,PEEK_* = 1.3 g/cm^3^) [49], and *n* is the scaling exponent.

Network stress and capillary pressure were estimated using the calculations of Smith, Scherer and Anderson [17]. Network stress is calculated as follows:

(12) $${\sigma_{x}=-P}_{y}+\frac{K_{0}}{n}\left(1-{\left(\frac{\rho }{\rho_{y}}\right)}^{n}\right)$$

where *σ_x_* indicates the maximum stress supported by the system, *K*_0_ is the bulk modulus of the gel before yield, *n* is the modulus–density scaling exponent, *ρ* is the aerogel bulk density, and *P_y_* is the pressure above which shrinkage is irreversible, determined as follows:

(13) $$P_{y}\equiv K_{0}ln\frac{\rho_{y}}{\rho }$$

and *ρ_y_* is the yield density, calculated as follows:

(14) $$\rho_{y}=\rho_{0}e^{\frac{1}{n}}$$

where *ρ*_0_ is the gel density prior to drying, estimated as follows:

(15) $$\rho_{0}=\frac{m}{V_{wet}}$$

and *K*_0_ was estimated from the compressive modulus using the following Equation:

(16) $$K_{0}=\frac{E_{0}}{3(1-2\nu )}$$

where ν is the Poisson’s ratio was assumed to be 0.2 [17] and *E*_0_ was the modulus at *ρ*_0_, calculated using Equation (11). The maximum pressure exerted on the gels during drying was calculated as follows:

(17) $$\sigma_{x}=\left(1-\frac{\rho }{\rho_{s}}\right)P_{c}$$

where *P_c_* is the capillary pressure:

(18) $$P_{c}=\frac{-2\gamma cos\theta }{r_{c}}$$

where *γ* is the surface tension, *θ* is the contact angle, and *r_c_* is the capillary radius. Surface tension of ethanol at 60 °C is 19 mN/m [50]. PEEK and PPS films for contact angle measurements were prepared on a Linkam (Salfords, UK) HFSX350-CAP temperature stage. The polymer was melted at 400 °C for PEEK or at 330 °C for PPS for at least 3 min and then pressed into a film. The films were cooled from the melt at 10 °C/min to room temperature. The contact angle of ethanol was found for PEEK and PPS with a Biolin Scientific (Gothenburg, Sweden) Theta Flow optical tensiometer using the sessile drop method (Figure S1). The contact angle of ethanol on the PEEK or PPS films was found to be 19.7° ± 5.6° and 17.3° ± 2.8°, respectively. The capillary radius, *r_c_*, was estimated as the hydraulic radius, defined as follows:

(19) $$r_{h}=\frac{2\left(1-\frac{\rho }{\rho_{s}}\right)}{{\rho A}_{s}}$$

where *A_s_* is the surface area of the dried gel. In this study, we assumed that surface area of a SC aerogel determined by the BET method was an acceptable substitution for *A_s_*. Equation (17) simplifies to the following:

(20) $$\sigma_{x}={\gamma cos\theta \rho A}_{s}$$

## Figures and Tables

**Table 1:** Calculated solution concentrations.

Polymer Solution	Polymer Concentration (wt%)	Polymer Concentration (vol%)
PEEK/DPA	10	8.6
PEEK/DPA	15	13.0
PEEK/DPA	20	17.5
PEEK/4CP	8.9	8.6
PEEK/4CP	13.4	13.0
PEEK/4CP	18.0	17.5
PPS/DPA	10	8.3
PPS/DPA	15	12.5
PPS/DPA	20	16.8

## Data Availability

Dataset available upon request from the authors.

## Supplementary Materials

The following supporting information can be downloaded at: https://www.mdpi.com/article/10.3390/gels11060447/s1, Figure S1. Representative sessile drop measurements of ethanol on a (a) PEEK or (b) PPS melt-pressed film. Figure S2. SEM micrographs of PEEK aerogels gelled from a 17.5vol.% PEEK/DPA solution and dried with (a,d) vacuum drying, (b,e) freeze drying, or (c,f) supercritical extraction. Images were collected at (a,b,c) 10 kx magnification or (d,e,f) 20 kx magnification. Figure S3. SEM micrographs of PEEK aerogels gelled from an 8.6vol.% PEEK/DPA solution and dried with (a,d) vacuum drying, (b,e) freeze drying, or (c,f) supercritical extraction. Images were collected at (a,b,c) 10 kx magnification or (d,e,f) 20 kx magnification. Figure S4. SEM micrographs of PEEK aerogels gelled from a 17.5vol.% PEEK/4CP solution and dried with (a,d) vacuum drying, (b,e) freeze drying, or (c,f) supercritical extraction. Images were collected at (a,b,c) 10 kx magnification or (d,e,f) 20 kx magnification. Figure S5. SEM micrographs of PEEK aerogels gelled from an 8.6vol.% PEEK/4CP solution and dried with (a,d) vacuum drying, (b,e) freeze drying, or (c,f) supercritical extraction. Images were collected at (a,b,c) 10 kx magnification or (d,e,f) 20 kx magnification. Figure S6. SEM micrographs of PPS aerogels gelled from a 16.8vol.% PPS/DPA solution and dried with (a,d) vacuum drying, (b,e) freeze drying, or (c,f) supercritical extraction. Images were collected at (a,b,c) 10 kx magnification or (d,e,f) 20 kx magnification. Figure S7. SEM micrographs of PPS aerogels gelled from an 8.3vol.% PPS/DPA solution and dried with (a,d) vacuum drying, (b,e) freeze drying, or (c,f) supercritical extraction. Images were collected at (a,b,c) 10 kx magnification or (d,e,f) 20 kx magnification. Figure S8. Merged USAXS/SAXS/WAXS profiles for aerogels prepared from solution compositions of (a) 13.0vol% PEEK/DPA, (b) 17.5vol% PEEK/DPA, (c) 13.0vol% PEEK/4CP, (d) 17.5vol% PEEK/4CP, (e) 12.5vol% PPS/DPA, and (f) 16.8vol% PPS/DPA. Profiles are vertically shifted for clarity. Table S1. Radius of Gyration (*R_g_*) and Porod Exponent (*P*) derived from application of the unified function to USAXS/SAXS profiles collected on PEEK or PPS aerogels. Table S2. Shrinkage, density, porosity, crystallinity, skeletal density, surface area, modulus, and yield stress of PEEK or PPS aerogels. Figure S9. PEEK gel cylinders prepared from 17.5 vol% PEEK/DPA solutions (a) imbibed with water, and after drying by (b) vacuum drying, (c) freeze drying, or (d) supercritical extraction. PEEK gel cylinders prepared from 17.5 vol% PEEK/4CP solutions (e) imbibed with water, and after drying by (f) vacuum drying, (g) freeze drying, or (h) supercritical extraction. Figure S10. (a) Shrinkage, (b) density, and (c) porosity of aerogel systems. Each aerogel systems is plotted together to aid comparison. Figure S11. Porosity of aerogels prepared from (a) PEEK/DPA solutions, (b) PEEK/4CP solutions, and (c) PPS/DPA solutions. Figure S12. Porosity versus density for polymer aerogels. Figure S13. Nitrogen sorption isotherms of aerogels prepared from solution compositions of (a) 13.0vol% PEEK/DPA, (b) 17.5vol% PEEK/DPA, (c) 13.0vol% PEEK/4CP, (d) 17.5vol% PEEK/4CP, (e) 12.5vol% PPS/DPA, and (f) 16.8vol% PPS/DPA. Figure S14. Pore size distribution of aerogels prepared from solution compositions of (a) 13.0vol% PEEK/DPA, (b) 17.5vol% PEEK/DPA, (c) 13.0vol% PEEK/4CP, (d) 17.5vol% PEEK/4CP, (e) 12.5vol% PPS/DPA, and (f) 16.8vol% PPS/DPA. Figure S15. Peak full width at half maximum (FWHM) of pore size distributions. Figure S16. Specific pore volume of aerogels prepared from (a) PEEK/DPA solutions, (b) PEEK/4CP solutions, and (c) PPS/DPA solutions. Table S3. Aerogel pore properties: specific pore volume, specific mesopore volume, specific macropore volume, and mesopore volume fraction. Figure S17. Aerogel pore properties: (a) specific pore volume, (b) specific mesopore volume, (c) specific macropore volume, and (d) mesopore volume fraction. Figure S18. Specific mesopore volume of aerogels prepared from (a) PEEK/DPA solutions, (b) PEEK/4CP solutions, and (c) PPS/DPA solutions. Figure S19. Specific macropore volume of aerogels prepared from (a) PEEK/DPA solutions, (b) PEEK/4CP solutions, and (c) PPS/DPA solutions. Figure S20. Mesopore volume fraction of aerogels prepared from (a) PEEK/DPA solutions, (b) PEEK/4CP solutions, and (c) PPS/DPA solutions. Figure S21. Compressive stress-strain curves for aerogels prepared from (a) an 8.6 vol.% PEEK/DPA solution, (b) a 13 vol.% PEEK/DPA solution, (c) a 17.5 vol.% PEEK/DPA solution, (d) an 8.6 vol.% PEEK/4CP solution, (e) a 13 vol.% PEEK/4CP solution, (f) a 17.5 vol.% PEEK/4CP solution, (g) an 8.3 vol.% PPS/DPA solution, (h) a 12.5 vol.% PPS/DPA solution, or (i) a 16.8 vol.% PPS/DPA solution, dried with different drying methods. Figure S22. Offset yield stress vs aerogel density for polymer aerogels. Figure S23. Network stress and capillary pressure vs density for (a) PEEK/DPA, (b) PEEK/4CP, or (c) PPS/DPA aerogels. Reference [51] is cited in the supplementary materials.

## References

[1] Flory P. (1974). Introductory lecture. Faraday Discuss. Chem. Soc..

[2] Nijenhuis K.t. (1997). Thermoreversible Networks: Viscoelastic Properties and Structure of Gels.

[3] Kim Y.S., Welch C.F., Hjelm R.P., Mack N.H., Labouriau A., Orler E.B. (2015). Origin of toughness in dispersion-cast Nafion membranes. Macromolecules.

[4] Shi S., Peng X., Liu T., Chen Y.-N., He C., Wang H. (2017). Facile preparation of hydrogen-bonded supramolecular polyvinyl alcohol-glycerol gels with excellent thermoplasticity and mechanical properties. Polymer.

[5] Matsuda H., Inoue T., Okabe M., Ukaji T. (1987). Study of polyolefin gel in organic solvents I. Structure of isotactic polypropylene gel in organic solvents. Polym. J..

[6] Daniel C., Menelle A., Brulet A., Guenet J.-M. (1997). Thermoreversible gelation of syndiotactic polystyrene in toluene and chloroform. Polymer.

[7] Xue G., Ji G., Li Y. (1998). Rapid crystallization and thermoreversible gelation of poly (ethylene terephthalate) in polymer/oligomer binary system. J. Polym. Sci. Part B Polym. Phys..

[8] Edwards C., Mandelkern L. (1982). Crystallization–gelation process of homopolymers and copolymers from solution. J. Polym. Sci. Polym. Lett. Ed..

[9] Mutin P., Guenet J. (1989). Physical gels from PVC: Aging and solvent effects on thermal behavior, swelling, and compression modulus. Macromolecules.

[10] Daniel C., Longo S., Fasano G., Vitillo J.G., Guerra G. (2011). Nanoporous Crystalline Phases of Poly(2,6-Dimethyl-1,4-phenylene)oxide. Chem. Mater..

[11] Spiering G.A., Godshall G.F., Moore R.B. (2024). High Modulus, Strut-like poly(ether ether ketone) Aerogels Produced from a Benign Solvent. Gels.

[12] Talley S.J., AndersonSchoepe C.L., Berger C.J., Leary K.A., Snyder S.A., Moore R.B. (2017). Mechanically robust and superhydrophobic aerogels of poly(ether ether ketone). Polymer.

[13] Talley S.J., Vivod S.L., Nguyen B.A., Meador M.A.B., Radulescu A., Moore R.B. (2019). Hierarchical Morphology of Poly(ether ether ketone) Aerogels. ACS Appl. Mater. Interfaces.

[14] Godshall G.F., Spiering G.A., Crater E.R., Moore R.B. (2023). Low-Density, Semicrystalline Poly(phenylene sulfide) Aerogels Fabricated Using a Benign Solvent. ACS Appl. Polym. Mater..

[15] Vareda J.P., Lamy-Mendes A., Durães L. (2018). A reconsideration on the definition of the term aerogel based on current drying trends. Microporous Mesoporous Mater..

[16] Smirnova I., Gurikov P. (2018). Aerogel production: Current status, research directions, and future opportunities. J. Supercrit. Fluids.

[17] Smith D., Scherer G., Anderson J. (1995). Shrinkage during drying of silica gel. J. Non-Cryst. Solids.

[18] Gurav J.L., Jung I.-K., Park H.-H., Kang E.S., Nadargi D.Y. (2010). Silica aerogel: Synthesis and applications. J. Nanomater..

[19] Di Luigi M., Guo Z., An L., Armstrong J.N., Zhou C., Ren S. (2022). Manufacturing silica aerogel and cryogel through ambient pressure and freeze drying. RSC Adv..

[20] Zhou T., Cheng X., Pan Y., Li C., Gong L., Zhang H. (2018). Mechanical performance and thermal stability of glass fiber reinforced silica aerogel composites based on co-precursor method by freeze drying. Appl. Surf. Sci..

[21] Scherer G.W. (1993). Freezing gels. J. Non-Cryst. Solids.

[22] Phalippou J., Woignier T., Prassas M. (1990). Glasses from aerogels: Part 1 The synthesis of monolithic silica aerogels. J. Mater. Sci..

[23] Schwan M., Nefzger S., Zoghi B., Oligschleger C., Milow B. (2021). Improvement of solvent exchange for supercritical dried aerogels. Front. Mater..

[24] Scherer G.W. (2019). Stress and strain during supercritical drying. J. Sol-Gel Sci. Technol..

[25] Ganesan K., Dennstedt A., Barowski A., Ratke L. (2016). Design of aerogels, cryogels and xerogels of cellulose with hierarchical porous structures. Mater. Des..

[26] Buchtová N., Budtova T. (2016). Cellulose aero-, cryo-and xerogels: Towards understanding of morphology control. Cellulose.

[27] Groult S., Buwalda S., Budtova T. (2021). Pectin hydrogels, aerogels, cryogels and xerogels: Influence of drying on structural and release properties. Eur. Polym. J..

[28] Job N., Théry A., Pirard R., Marien J., Kocon L., Rouzaud J.-N., Béguin F., Pirard J.-P. (2005). Carbon aerogels, cryogels and xerogels: Influence of the drying method on the textural properties of porous carbon materials. Carbon.

[29] Ciftci D., Ubeyitogullari A., Huerta R.R., Ciftci O.N., Flores R.A., Saldaña M.D. (2017). Lupin hull cellulose nanofiber aerogel preparation by supercritical CO_2_ and freeze drying. J. Supercrit. Fluids.

[30] Guastaferro M., Baldino L., Reverchon E., Cardea S. (2021). Production of porous agarose-based structures: Freeze-drying vs. supercritical CO_2_ drying. Gels.

[31] Buckley A., Greenblatt M. (1992). A comparison of the microstructural properties of silica aerogels and xerogels. J. Non-Cryst. Solids.

[32] Brock S.L., Arachchige I.U., Kalebaila K.K. (2006). Metal chalcogenide gels, xerogels and aerogels. Comments Inorg. Chem..

[33] Fougnies C., Damman P., Dosiere M., Koch M. (1997). Time-resolved SAXS, WAXS, and DSC study of melting of poly (aryl ether ether ketone)(PEEK) annealed from the amorphous state. Macromolecules.

[34] Hay J., Langford J., Lloyd J. (1989). Variation in unit cell parameters of aromatic polymers with crystallization temperature. Polymer.

[35] Tabor B., Magre E., Boon J. (1971). The crystal structure of poly-p-phenylene sulphide. Eur. Polym. J..

[36] Napolitano R., Pirozzi B., Salvione A. (1999). Crystal structure of poly (p-phenylene sulfide): A refinement by X-ray measurements and molecular mechanics calculations. Macromolecules.

[37] Beaucage G. (1995). Approximations leading to a unified exponential/power-law approach to small-angle scattering. J. Appl. Crystallogr..

[38] Bisson A., Rigacci A., Lecomte D., Rodier E., Achard P. (2003). Drying of silica gels to obtain aerogels: Phenomenology and basic techniques. Dry. Technol..

[39] Thommes M., Kaneko K., Neimark A.V., Olivier J.P., Rodriguez-Reinoso F., Rouquerol J., Sing K.S.W. (2015). Physisorption of gases, with special reference to the evaluation of surface area and pore size distribution (IUPAC Technical Report). Pure Appl. Chem..

[40] Baldovino-Medrano V.c.G., Niño-Celis V., Isaacs Giraldo R. (2023). Systematic analysis of the nitrogen adsorption–desorption isotherms recorded for a series of materials based on microporous–mesoporous amorphous aluminosilicates using classical methods. J. Chem. Eng. Data.

[41] Bardestani R., Patience G.S., Kaliaguine S. (2019). Experimental methods in chemical engineering: Specific surface area and pore size distribution measurements—BET, BJH, and DFT. Can. J. Chem. Eng..

[42] Brunauer S., Emmett P.H., Teller E. (1938). Adsorption of gases in multimolecular layers. J. Am. Chem. Soc..

[43] Gibson I., Ashby M.F. (1982). The mechanics of three-dimensional cellular materials. Proc. R. Soc. Lond. A. Math. Phys. Sci..

[44] Ma H.-S., Roberts A.P., Prévost J.-H., Jullien R., Scherer G.W. (2000). Mechanical structure–property relationship of aerogels. J. Non-Cryst. Solids.

[45] Blundell D.J., Osborn B.N. (1983). The morphology of poly (aryl-ether-ether-ketone). Polymer.

[46] Hay J.N., Luck D.A. (2001). The conformation of crystalline poly(phenylene sulphide). Polymer.

[47] Ilavsky J., Jemian P.R. (2009). Irena: Tool suite for modeling and analysis of small-angle scattering. J. Appl. Crystallogr..

[48] (2023). Standard Test Method for Compressive Properties of Rigid Plastics.

[49] Nikonovich M., Costa J.F.S., Fonseca A.C., Ramalho A., Emami N. (2023). Structural, thermal, and mechanical characterisation of PEEK-based composites in cryogenic temperature. Polym. Test..

[50] Vogel A.I. (1948). 366. Physical properties and chemical constitution. Part XX. Aliphatic alcohols and acids. J. Chem. Soc. (Resumed).

[51] Roe R.-J. (2000). Methods of X-Ray and Neutron Scattering in Polymer Science.

