# Supplementary material for: Effect of the Gel Drying Method on Properties of Semicrystalline Aerogels Prepared with Different Network Morphologies
Source: Gels. 2025 Jun 10;11(6):447. doi: 10.3390/gels11060447 (PMC12191750; doi:10.3390/gels11060447)
Supplement: Supplementary file 1 [file gels-11-00447-s001.zip › gels-3683479-supplementary.pdf]

Supporting Information

Effect of Drying Method on Aerogel Properties in Semicrystalline Aerogels Prepared with Different Network Morphologies

Glenn A. Spiering, Garrett F. Godshall, Robert B. Moore*

Macromolecules Innovation Institute and Department of Chemistry, Virginia Polytechnic Institute and State University, Blacksburg, VA 24061, USA

*rbmoore3@vt.edu

Keywords: aerogel; semicrystalline polymer aerogel; aerogel drying; vacuum drying; freeze drying; supercritical drying; hierarchical morphology

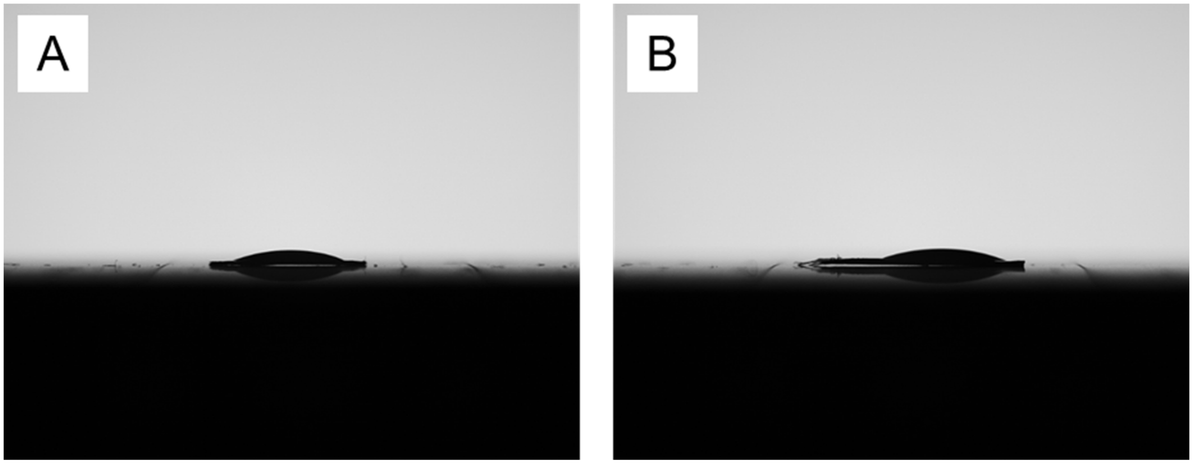

Figure S1. Representative sessile drop measurements of ethanol on a (a) PEEK or (b) PPS melt-pressed film.

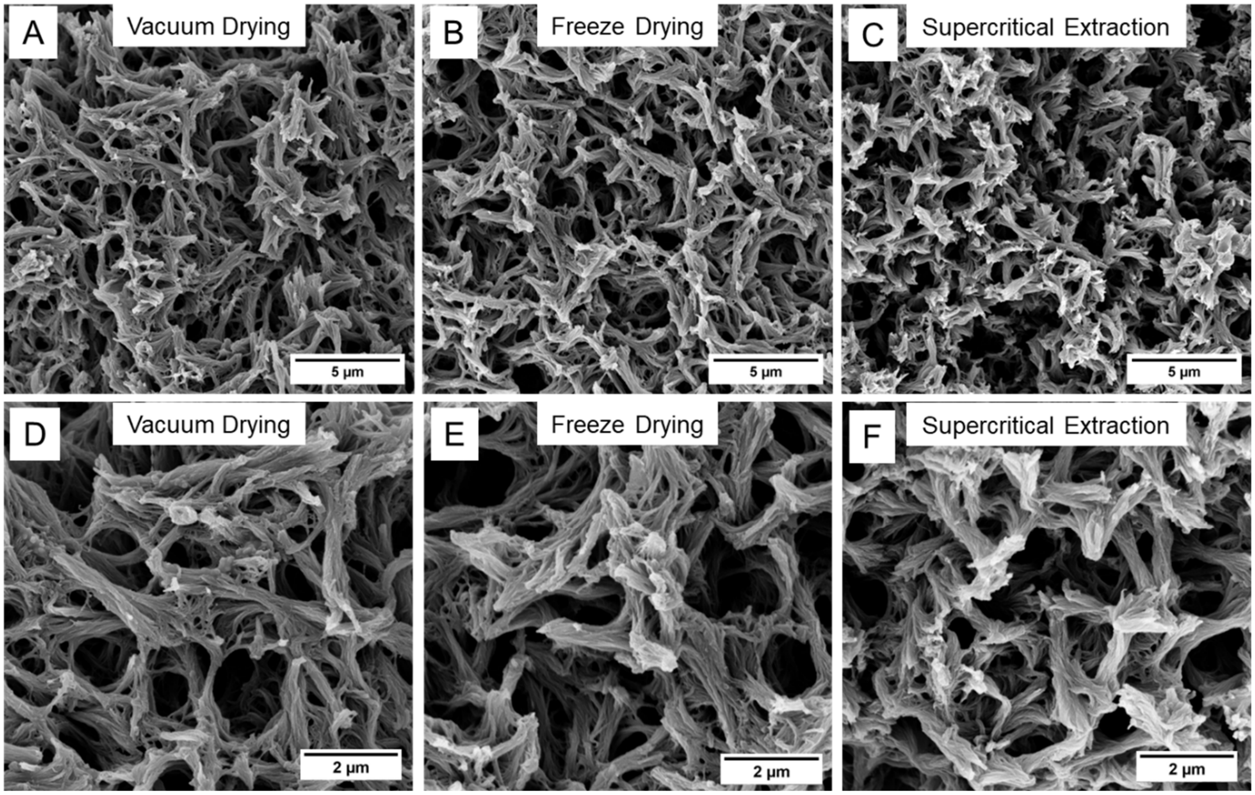

Figure S2. SEM micrographs of PEEK aerogels gelled from a 17.5vol.% PEEK/DPA solution and dried with (a,d) vacuum drying, (b,e) freeze drying, or (c,f) supercritical extraction. Images were collected at (a,b,c) 10 kx magnification or (d,e,f) 20 kx magnification.

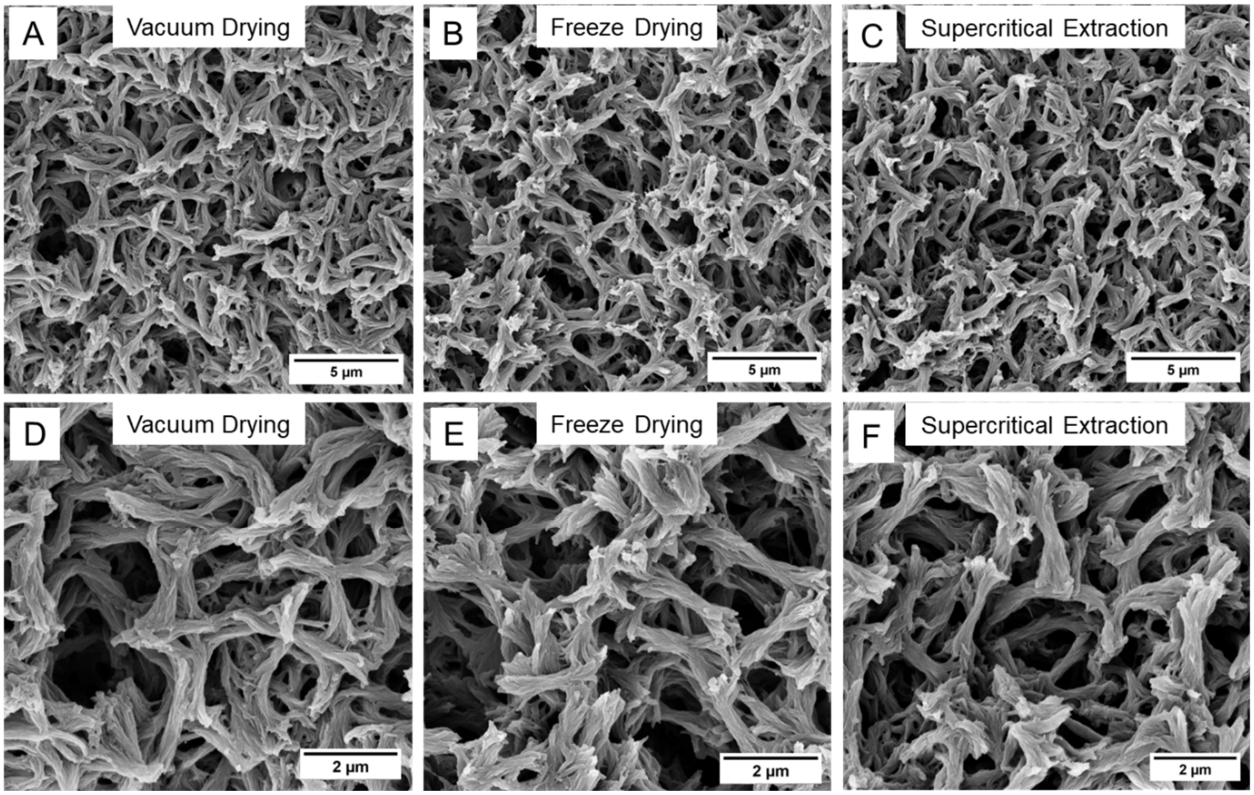

Figure S3. SEM micrographs of PEEK aerogels gelled from an 8.6vol.% PEEK/DPA solution and dried with (a,d) vacuum drying, (b,e) freeze drying, or (c,f) supercritical extraction. Images were collected at (a,b,c) 10 kx magnification or (d,e,f) 20 kx magnification.

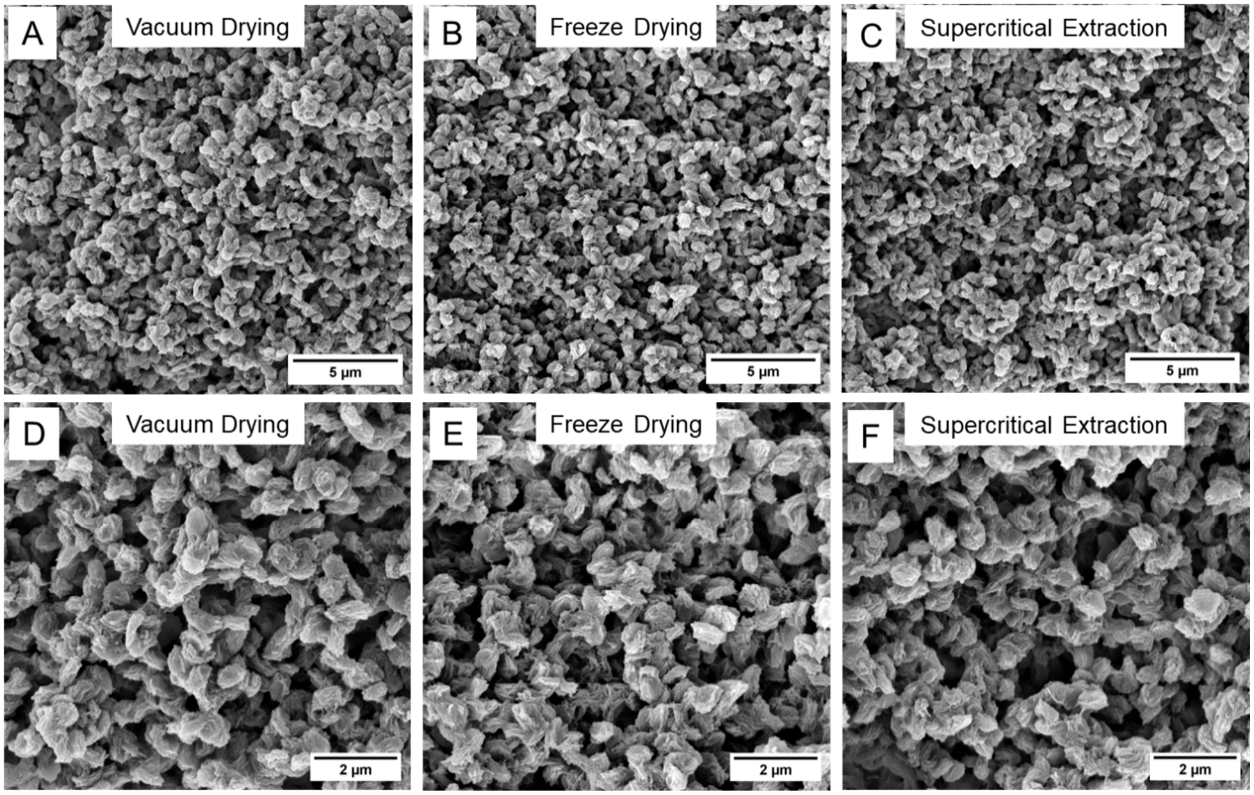

Figure S4. SEM micrographs of PEEK aerogels gelled from a 17.5vol.% PEEK/4CP solution and dried with (a,d) vacuum drying, (b,e) freeze drying, or (c,f) supercritical extraction. Images were collected at (a,b,c) 10 kx magnification or (d,e,f) 20 kx magnification.

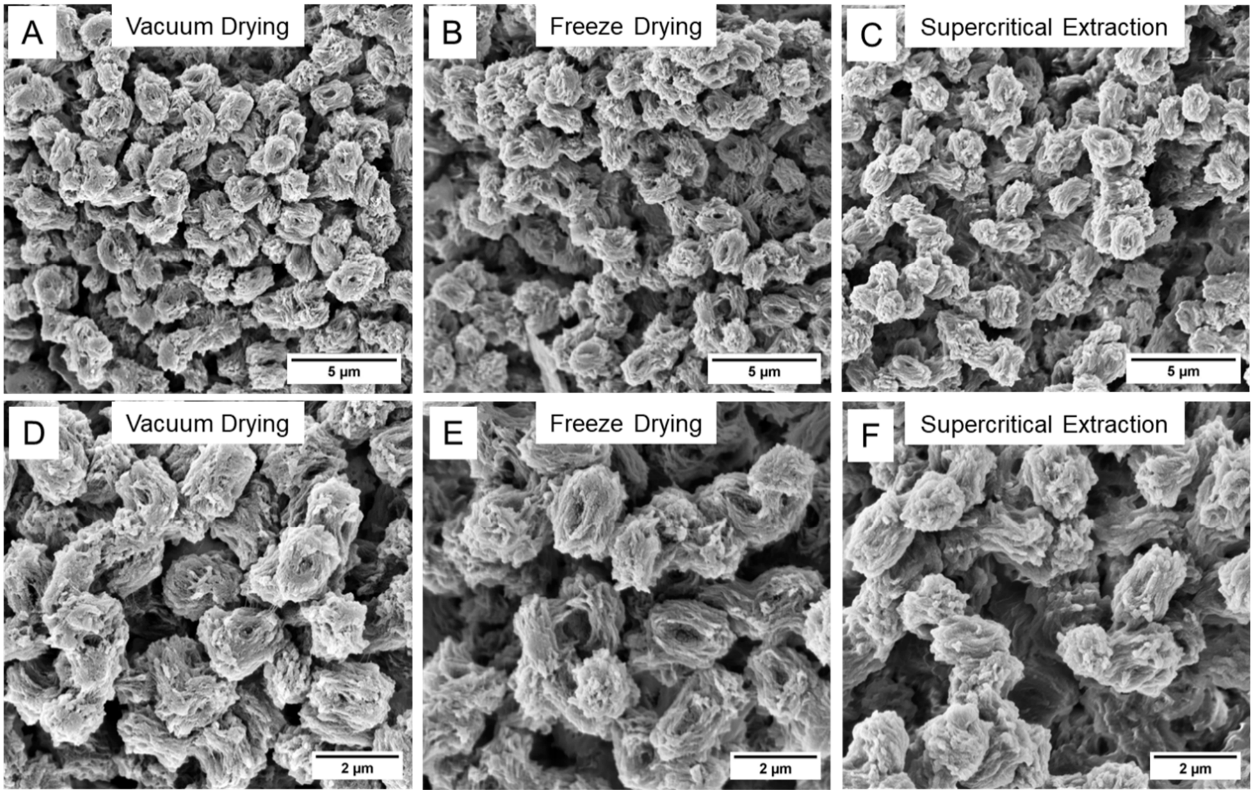

Figure S5. SEM micrographs of PEEK aerogels gelled from an 8.6vol.% PEEK/4CP solution and dried with (a,d) vacuum drying, (b,e) freeze drying, or (c,f) supercritical extraction. Images were collected at (a,b,c) 10 kx magnification or (d,e,f) 20 kx magnification.

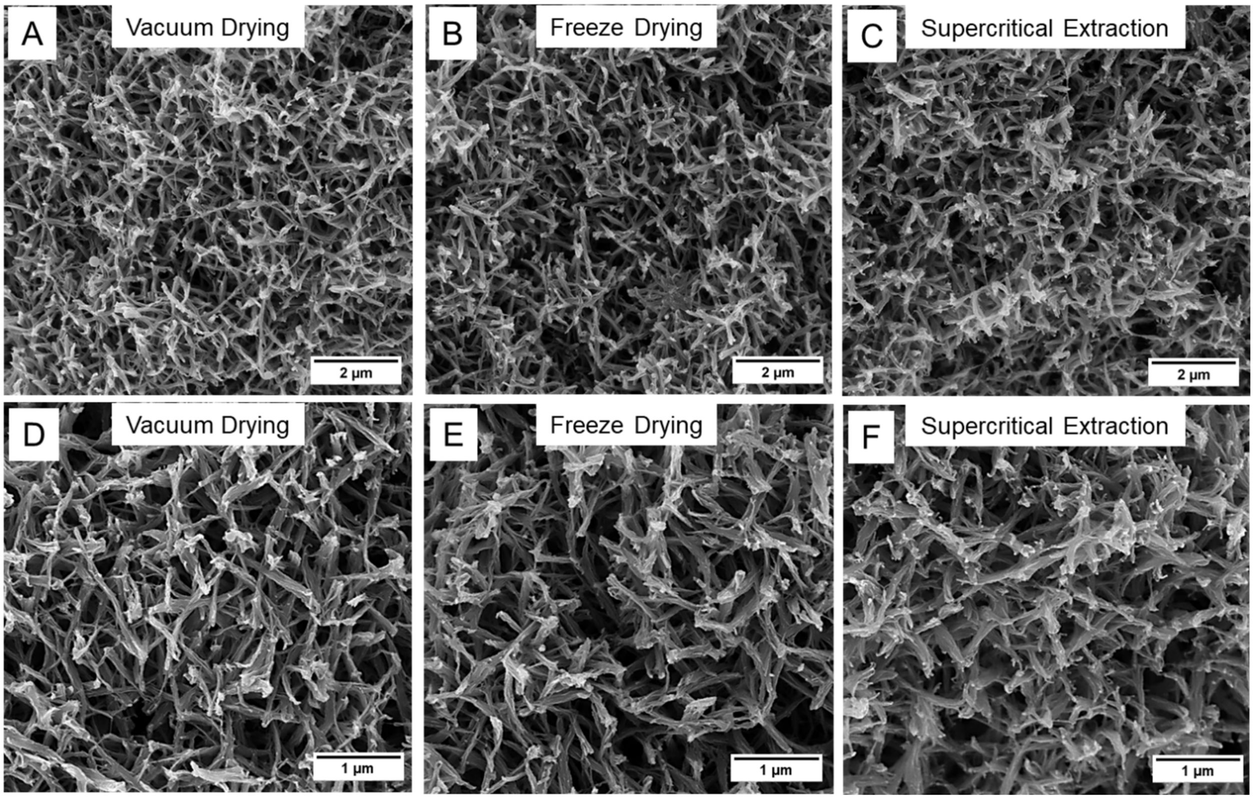

Figure S6. SEM micrographs of PPS aerogels gelled from a 16.8vol.% PPS/DPA solution and dried with (a,d) vacuum drying, (b,e) freeze drying, or (c,f) supercritical extraction. Images were collected at (a,b,c) 10 kx magnification or (d,e,f) 20 kx magnification.

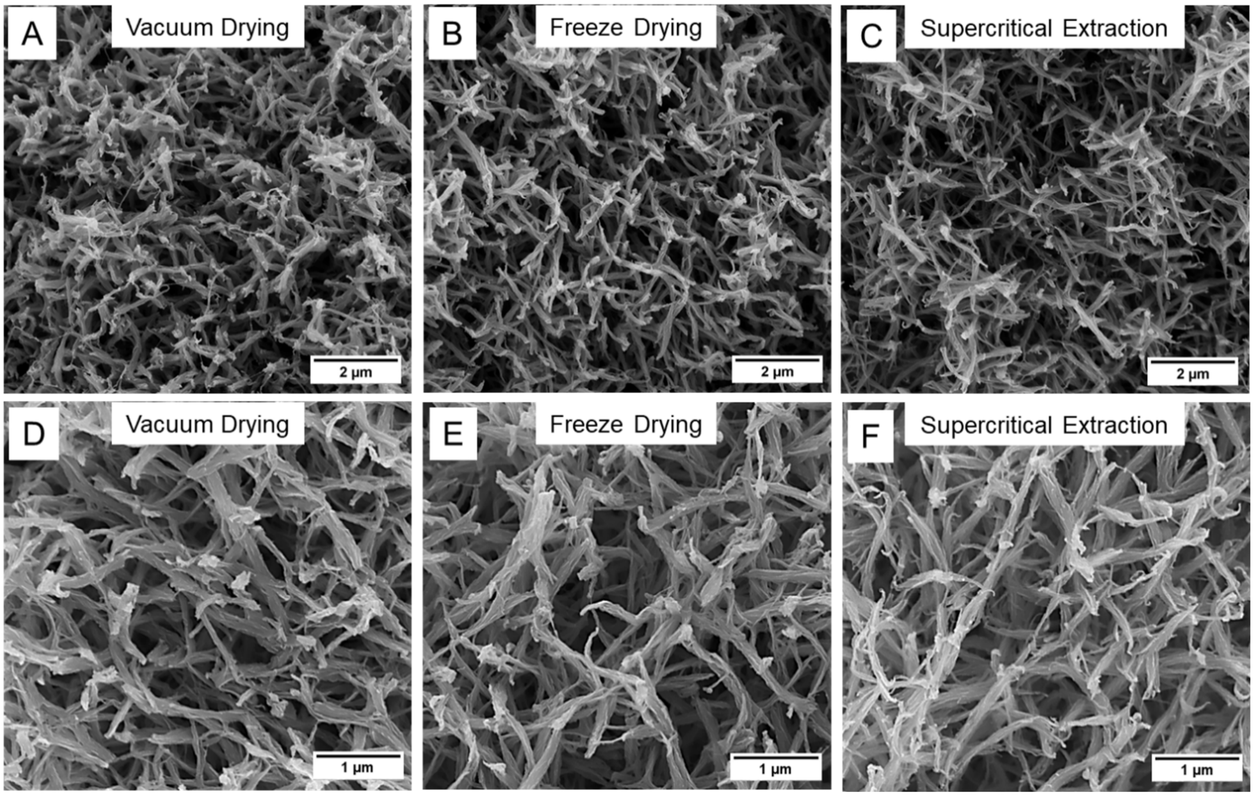

Figure S7. SEM micrographs of PPS aerogels gelled from an 8.3vol.% PPS/DPA solution and dried with (a,d) vacuum drying, (b,e) freeze drying, or (c,f) supercritical extraction. Images were collected at (a,b,c) 10 kx magnification or (d,e,f) 20 kx magnification.

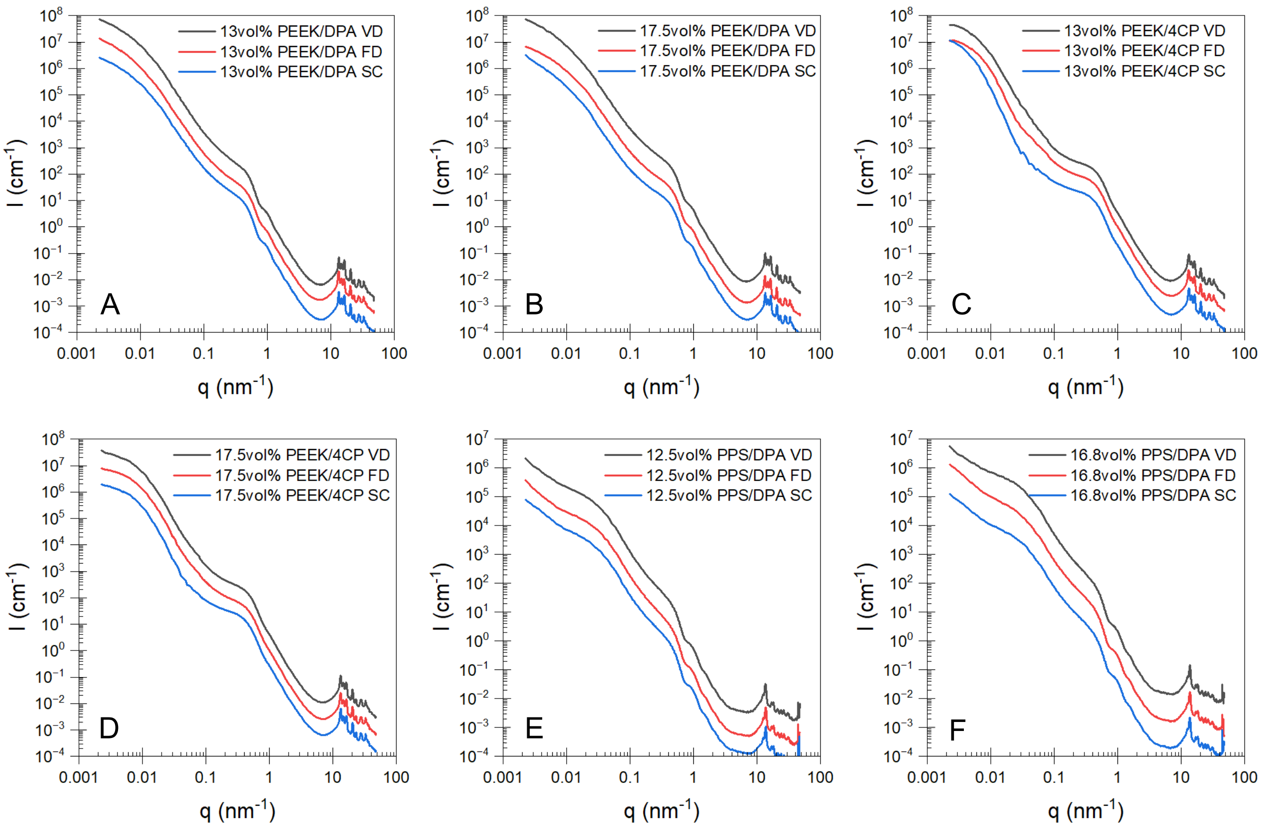

Figure S8. Merged USAXS/SAXS/WAXS profiles for aerogels prepared from solution compositions of (a) 13.0vol% PEEK/DPA, (b) 17.5vol% PEEK/DPA, (c) 13.0vol% PEEK/4CP, (d) 17.5vol% PEEK/4CP, (e) 12.5vol% PPS/DPA, and (f) 16.8vol% PPS/DPA. Profiles are vertically shifted for clarity.

X-ray Scattering Analysis

The scattering patterns for all aerogels prepared with similar drying methods from PEEK/DPA (**Figure 5a**, **S8a**, and **S8b**) or PPS/DPA (**Figure 5c**, **S8e** and **S8f**) solutions display similar profiles across the scattering vectors probed experimentally. Generally, the scattering profiles for the PEEK/4CP (**Figure 5b**, **S8c** and **S8d**) system appear comparable between aerogels prepared from different drying methods. However, differences between aerogels prepared with different drying methods were observed in the power law regime between 0.01 nm^{-1} and 0.1 nm^{-1}.

The power law slope is more negative for samples prepared with supercritical extraction, then became more positive for freeze dried samples, then was even more positive for vacuum dried samples. Changes in the power law slope indicate a change in the fractality, or roughness of the physical feature. Quantification of the scattering patterns will allow more informed analysis of the scattering features.

In order to quantify the hierarchical structure of semicrystalline polymer aerogels, merged USAXS/SAXS/WAXS profiles were fit to the unified equation.[1] The unified equation allows a hierarchical scattering pattern to be described using multiple Guinier and Porod features, which describe the size and fractality of scattering features, respectively. For each structural level, the feature size is given by the radius of gyration, R_g, and the fractality of the interface is quantified by the Porod exponent, P. Feature 1 is assigned to the crystallite feature observed as a knee between 0.2 nm^{-1} and 0.8 nm^{-1} and feature 2 is assigned to the larger aggregates observed as the knee below 0.1 nm^{-1}. Results of fitting can be seen in **Table S1**.

For the fibrillar aerogels prepared from PEEK/DPA or PPS/DPA solutions, the size of the aggregate feature, R_{g2}, generally stays constant at 200 nm or 60 nm, respectively. The size of the aggregate feature for the PPS aerogels is smaller than this feature for PEEK/DPA aerogels, in good agreement with the SEM images (**Figures 2 and 4**). No significant influence of drying method on aggregate size is observed for the fibrillar aerogels. For both systems, the power law region following the aggregate feature has a Porod exponent P_2 between 3 and 4, indicating that the surface of the aggregates can be described as a surface fractal.[2] For fibrillar aerogels, the size of the crystallite feature, R_{g1}, is found with dimensions between 5-6 nm. The Porod exponent P_1 is 4 for each, indicating smooth interfaces, which is expected for the surfaces of polymer crystals.[2] No significant trends were observed in R_{g1}, P_2, or P_1 with respect to drying technique.

The scattering profiles of aerogels prepared from PEEK/4CP were also fit with the unified equation. The radius of gyration of the aggregate feature, R_{g2}, tends to slightly larger values with decreasing the aggressiveness of the drying method, from vacuum drying to freeze drying to supercritical drying. This trend indicates the shrinkage of the PEEK aggregate features that make up the physical network. Aerogels prepared at 8.6 and 13 vol% have larger aggregate features than the 17.5 vol%, in agreement with the SEM images (**Fig. 3, S4, S5**). The Porod exponent P_2 tends to increase with decreasing aggressiveness of the drying method, from vacuum drying to freeze drying to supercritical drying. Increases in the power-law slope indicate an increase in the fractal dimension, meaning that the surface of the feature displays a rougher interface.[2] Surface roughness is implied to increase with increasing the aggressiveness of the drying method. For vacuum drying, capillary forces acting on the fine mesopores coating the surface of the aggregates disrupt this structure, and therefore affect the surface texture of the dried gel.[3] Prior to freeze drying, the freezing of ice within a gel can also affect pore morphology as the growth of ice crystals is known to exert force on the gel network.[4] The size of the crystallite feature R_{g1} is smaller than the PEEK/DPA system, with values between 5 and 4.5 nm. Values for R_{g1} tend to be highest for gels prepared by supercritical drying and lowest for samples prepared by vacuum drying. The more aggressive drying methods appear to distort these crystallite features. P_1 values are 4, again indicating smooth surfaces.[2]

Table S1. Radius of Gyration (R_g) and Porod Exponent (P) derived from application of the unified function to USAXS/SAXS profiles collected on PEEK or PPS aerogels.

Aerogel	Rg2 (nm)	P2	Rg1 (nm)	P1
8.6vol% PEEK/DPA VD	203	3.68	5.05	4.0
8.6vol% PEEK/DPA FD	203	3.66	5.56	4.0
8.6vol% PEEK/DPA SC	221	3.72	5.33	4.0
13vol% PEEK/DPA VD	152	3.76	5.34	4.0
13vol% PEEK/DPA FD	192	3.59	5.36	4.0
13vol% PEEK/DPA SC	199	3.84	5.86	4.0
17.5vol% PEEK/DPA VD	192	3.71	5.39	4.0
17.5vol% PEEK/DPA FD	195	3.57	5.40	4.0
17.5vol% PEEK/DPA SC	198	3.36	5.45	4.0
8.6vol% PEEK/4CP VD	312	3.59	4.49	4.0
8.6vol% PEEK/4CP FD	383	3.81	4.89	4.0
8.6vol% PEEK/4CP SC	453	4.50	4.99	4.0
13vol% PEEK/4CP VD	362	3.75	4.61	4.0
13vol% PEEK/4CP FD	337	4.08	4.77	4.0
13vol% PEEK/4CP SC	492	4.59	5.00	4.0
17.5vol% PEEK/4CP VD	263	3.89	4.76	4.0
17.5vol% PEEK/4CP FD	263	4.09	4.85	4.0
17.5vol% PEEK/4CP SC	270	4.24	4.89	4.0
8.3vol% PPS/DPA VD	62.6	3.89	5.64	4.0
8.3vol% PPS/DPA FD	67.8	3.58	5.51	4.0
8.3vol% PPS/DPA SC	65.4	4.00	5.65	4.0
12.5vol% PPS/DPA VD	64.4	3.86	5.74	4.0
12.5vol% PPS/DPA FD	65.7	3.71	5.68	4.0
12.5vol% PPS/DPA SC	52.5	3.83	6.04	4.0
16.8vol% PPS/DPA VD	60.6	3.67	5.60	4.0
16.8vol% PPS/DPA FD	53.5	3.70	5.88	4.0
16.8vol% PPS/DPA SC	52.2	3.49	5.96	4.0

Table S2. Shrinkage, density, porosity, crystallinity, skeletal density, surface area, modulus, and yield stress of PEEK or PPS aerogels.

Gel System	Drying method	Shrinkage (%)	Density (g/cm^3)	Porosity (%)	Crystallinity (%)	Skeletal Density (g/cm^3)	Surface Area (m^2/g)	Modulus (MPa)	Yield Stress (MPa)
8.6vol% PEEK/DPA	Vacuum Drying	20.3 ± 1.2	0.138 ± 0.001	89.7 ± 2.7	55.6 ± 2.0	1.34 ± 0.04	170 ± 17	11.00 ± 1.64	0.231 ± 0.040
8.6vol% PEEK/DPA	Freeze Drying	6.1 ± 1.3	0.117 ± 0.002	91.3 ± 6.0	52.3 ± 4.5	1.33 ± 0.09	187 ± 23	5.87 ± 0.55	0.138 ± 0.022
8.6vol% PEEK/DPA	Supercritical Extraction	1.7 ± 0.1	0.111 ± 0.001	91.7 ± 3.6	54.6 ± 2.8	1.34 ± 0.05	206 ± 20	3.56 ± 0.16	0.081 ± 0.005
13vol% PEEK/DPA	Vacuum Drying	7.4 ± 0.5	0.172 ± 0.002	87.1 ± 1.0	54.1 ± 0.4	1.34 ± 0.01	178 ± 6	19.33 ± 0.35	0.610 ± 0.026
13vol% PEEK/DPA	Freeze Drying	4.2 ± 0.4	0.167 ± 0.001	87.5 ± 0.6	55.6 ± 0.3	1.34 ± 0.01	190 ± 13	19.19 ± 1.77	0.425 ± 0.048
13vol% PEEK/DPA	Supercritical Extraction	3.0 ± 0.7	0.165 ± 0.001	87.7 ± 0.9	54.2 ± 0.5	1.34 ± 0.01	208 ± 9	14.23 ± 2.53	0.318 ± 0.015
17.5vol% PEEK/DPA	Vacuum Drying	7.1 ± 0.7	0.225 ± 0.003	83.2 ± 2.7	56.5 ± 2.2	1.34 ± 0.04	174 ± 4	50.09 ± 5.09	1.173 ± 0.075
17.5vol% PEEK/DPA	Freeze Drying	4.6 ± 0.5	0.222 ± 0.002	83.4 ± 4.4	53.0 ± 3.7	1.34 ± 0.07	183 ± 7	44.48 ± 0.83	0.990 ± 0.113
17.5vol% PEEK/DPA	Supercritical Extraction	3.8 ± 0.5	0.216 ± 0.001	83.9 ± 1.3	54.9 ± 1.1	1.34 ± 0.02	192 ± 11	31.14 ± 8.69	0.779 ± 0.019
8.6vol% PEEK/4CP	Vacuum Drying	25.4 ± 0.9	0.179 ± 0.005	86.6 ± 3.0	56.0 ± 1.5	1.34 ± 0.03	234 ± 1	3.40 ± 0.58	0.101 ± 0.010
8.6vol% PEEK/4CP	Freeze Drying	9.0 ± 0.4	0.151 ± 0.003	88.8 ± 5.0	61.3 ± 3.8	1.35 ± 0.07	275 ± 3	0.89 ± 0.10	0.022 ± 0.004
8.6vol% PEEK/4CP	Supercritical Extraction	13.9 ± 2.5	0.157 ± 0.003	88.3 ± 4.0	58.9 ± 2.9	1.34 ± 0.05	297 ± 4	1.03 ± 0.13	0.023 ± 0.007
13vol% PEEK/4CP	Vacuum Drying	27.4 ± 1.3	0.289 ± 0.000	78.4 ± 2.1	56.8 ± 1.9	1.34 ±0.04	211 ± 10	13.48 ± 1.81	0.460 ± 0.027

13vol% PEEK/4CP	Freeze Drying	11.6 ± 0.5	0.237 ± 0.003	82.3 ± 3.9	53.5 ± 3.3	1.34 ± 0.06	247 ± 13	5.12 ± 0.41	0.110 ± 0.007
13vol% PEEK/4CP	Supercritical Extraction	13.9 ± 0.7	0.243 ± 0.005	81.9 ± 2.1	57.1 ± 1.1	1.34 ± 0.02	281 ± 8	5.83 ± 0.38	0.129 ± 0.016
17.5vol% PEEK/4CP	Vacuum Drying	31.2 ± 2.1	0.406 ± 0.009	69.7 ± 2.0	56.9 ± 1.4	1.34 ± 0.03	219 ± 4	62.57 ± 12.15	1.576 ± 0.082
17.5vol% PEEK/4CP	Freeze Drying	18.0 ± 0.4	0.344 ± 0.003	74.3 ± 2.1	55.8 ± 1.9	1.34 ± 0.04	225 ± 9	32.86 ± 8.00	0.890 ± 0.261
17.5vol% PEEK/4CP	Supercritical Extraction	11.9 ± 0.7	0.322 ± 0.003	76.0 ± 1.2	56.7 ± 0.9	1.34 ± 0.02	276 ± 6	21.71 ± 3.87	0.479 ± 0.078
8.3vol% PPS/DPA	Vacuum Drying	39.8 ± 1.3	0.189 ± 0.001	84.8 ± 0.2	-	1.24 ± 0.02	117 ± 7	16.26 ± 0.98	0.651 ± 0.151
8.3vol% PPS/DPA	Freeze Drying	4.0 ± 1.0	0.122 ± 0.004	90.7 ± 0.5	-	1.31 ± 0.06	119 ± 4	5.74 ± 0.91	0.197 ± 0.017
8.3vol% PPS/DPA	Supercritical Extraction	1.9 ± 2.2	0.112 ± 0.003	90.9 ± 0.3	-	1.23 ± 0.01	125 ± 2	3.81 ± 0.05	0.136 ± 0.043
12.5vol% PPS/DPA	Vacuum Drying	4.5 ± 0.8	0.173 ± 0.000	86.1 ± 0.3	-	1.24 ± 0.03	119 ± 1	20.50 ± 0.81	0.674 ± 0.018
12.5vol% PPS/DPA	Freeze Drying	1.8 ± 0.3	0.169 ± 0.002	86.3 ± 0.3	-	1.24 ± 0.03	117 ± 4	16.11 ± 0.70	0.631 ± 0.039
12.5vol% PPS/DPA	Supercritical Extraction	0.5 ± 0.9	0.167 ± 0.001	86.7 ± 0.1	-	1.25 ± 0.00	116 ± 1	13.39 ± 0.43	0.492 ± 0.007
16.8vol% PPS/DPA	Vacuum Drying	1.5 ± 1.2	0.230 ± 0.002	81.9 ± 0.0	-	1.27 ± 0.01	112 ± 3	35.96 ± 3.95	1.321 ± 0.018
16.8vol% PPS/DPA	Freeze Drying	1.4 ± 1.2	0.226 ± 0.004	82.2 ± 0.1	-	1.27 ± 0.01	117 ± 1	35.95 ± 3.86	1.336 ± 0.059
16.8vol% PPS/DPA	Supercritical Extraction	0.6 ± 0.7	0.224 ± 0.001	82.2 ± 0.1	-	1.26 ± 0.00	111 ± 1	31.58 ± 2.39	1.191 ± 0.064

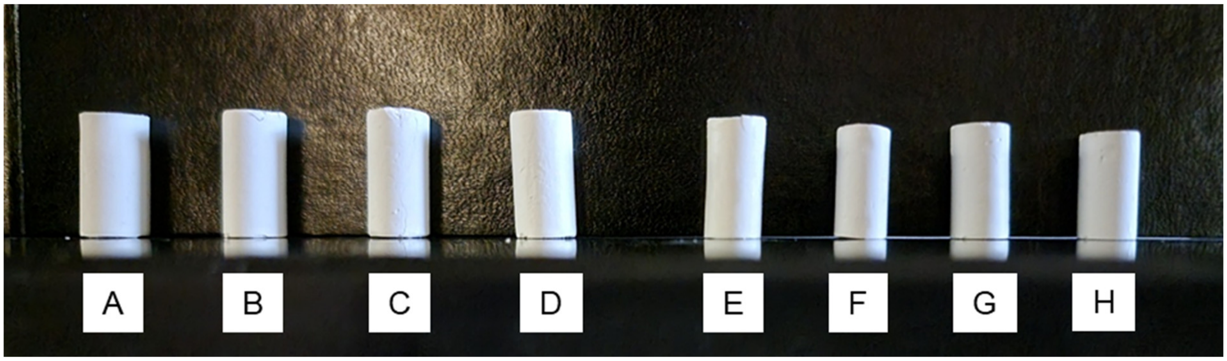

Figure S9. PEEK gel cylinders prepared from 17.5 vol% PEEK/DPA solutions (a) imbibed with water, and after drying by (b) vacuum drying, (c) freeze drying, or (d) supercritical extraction. PEEK gel cylinders prepared from 17.5 vol% PEEK/4CP solutions (e) imbibed with water, and after drying by (f) vacuum drying, (g) freeze drying, or (h) supercritical extraction.

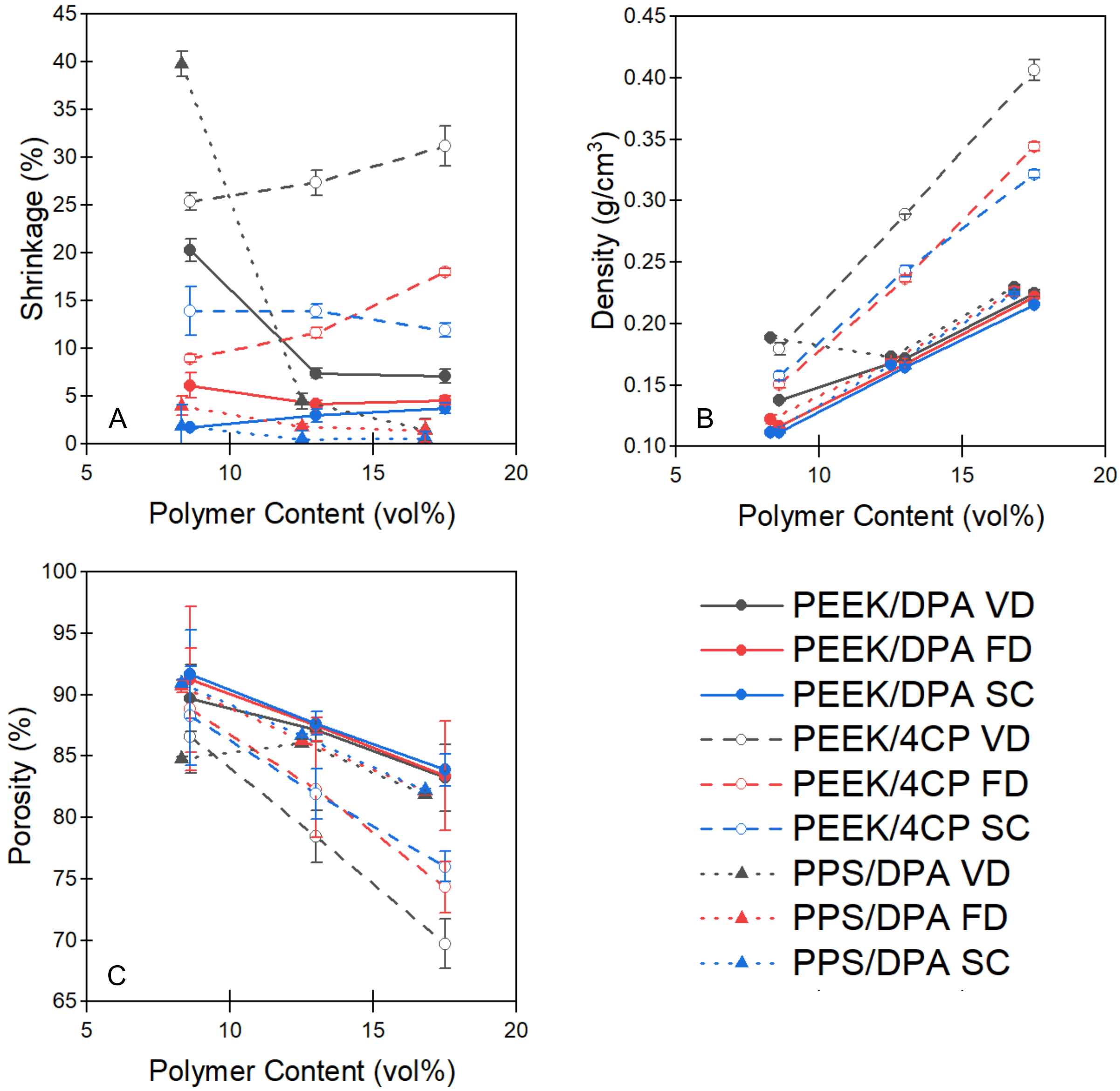

Figure S10. (a) Shrinkage, (b) density, and (c) porosity of aerogel systems. Each aerogel systems is plotted together to aid comparison.

Porosity analysis

Shrinkage of an aerogel during solvent extraction also undesirably decreases the requisite porosity of an aerogel. **Figure S11a**, **S11b**, or **S11c** show the porosity of aerogels derived from PEEK/DPA solutions, PEEK/4CP solutions, or PPS/DPA solutions, respectively. Tabulated porosity values can be seen in **Table S2**. For each of the aerogel systems, porosity generally tends to decrease linearly with increasing polymer content. The similar strut-like morphologies of the PEEK/DPA and PPS/DPA aerogels yield similar decreases in porosity with polymer content and relatively high porosities compared to the globular PEEK/4CP aerogels. With respect to drying method, vacuum dried aerogels tend to have the lowest porosity, followed by freeze-dried aerogels, and then aerogels that were extracted with supercritical fluid. With the more aggressive drying methods of vacuum drying and freeze-drying, shrinkage decreases the open volume between the structural elements of the aerogel framework, thereby decreasing the open porosity. The overall lower porosity of the PEEK/4CP aerogels and the greater sensitivity to drying method is attributed to their globular morphology (compared to the strut-like morphologies of the aerogels prepared from DPA). Apparently, the globular features are more loosely interconnected and easier to pack into more dense networks as polymer content and/or aggressiveness of the drying method increase. Finally, when considering the final density of the processed aerogels, the porosities of all the aerogels collapse onto a single linear relationship between porosity and density (**Figure S12**).

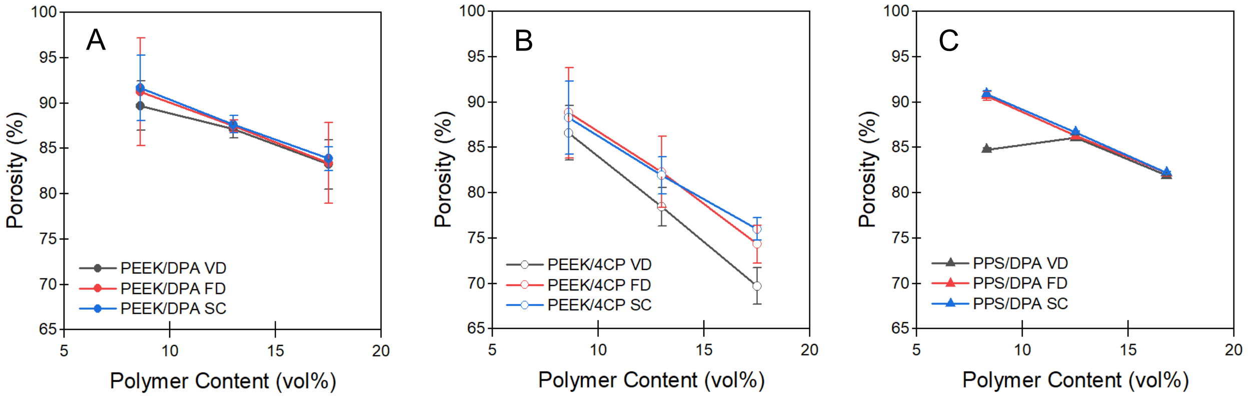

Figure S11. Porosity of aerogels prepared from (a) PEEK/DPA solutions, (b) PEEK/4CP solutions, and (c) PPS/DPA solutions.

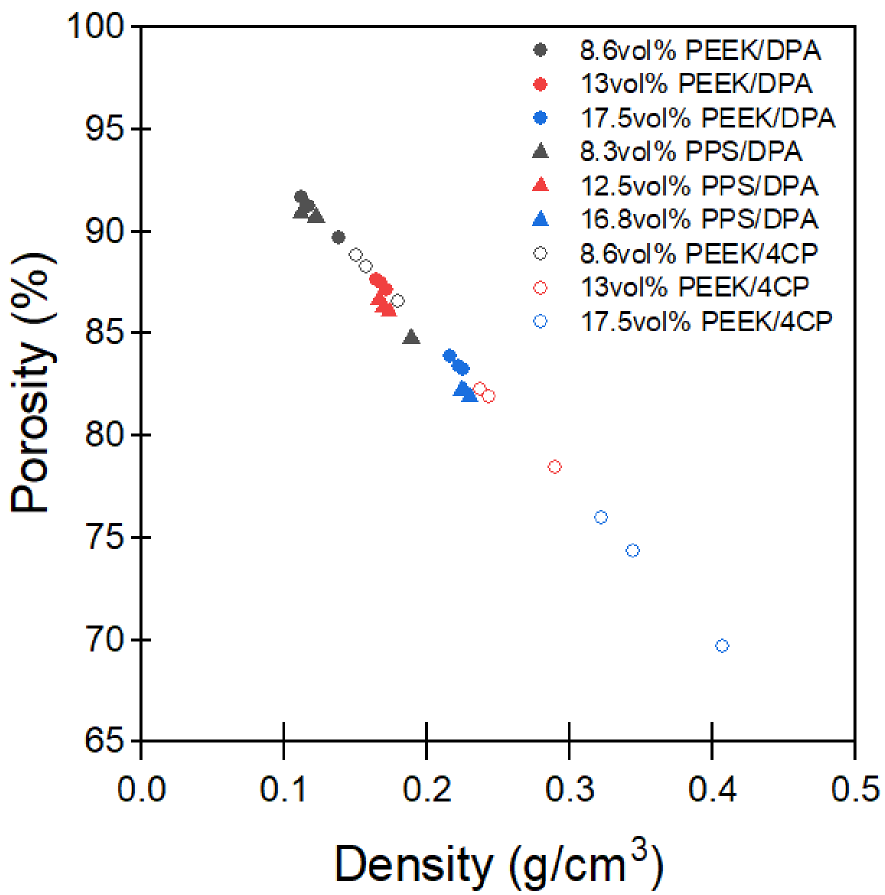

Figure S12. Porosity versus density for polymer aerogels

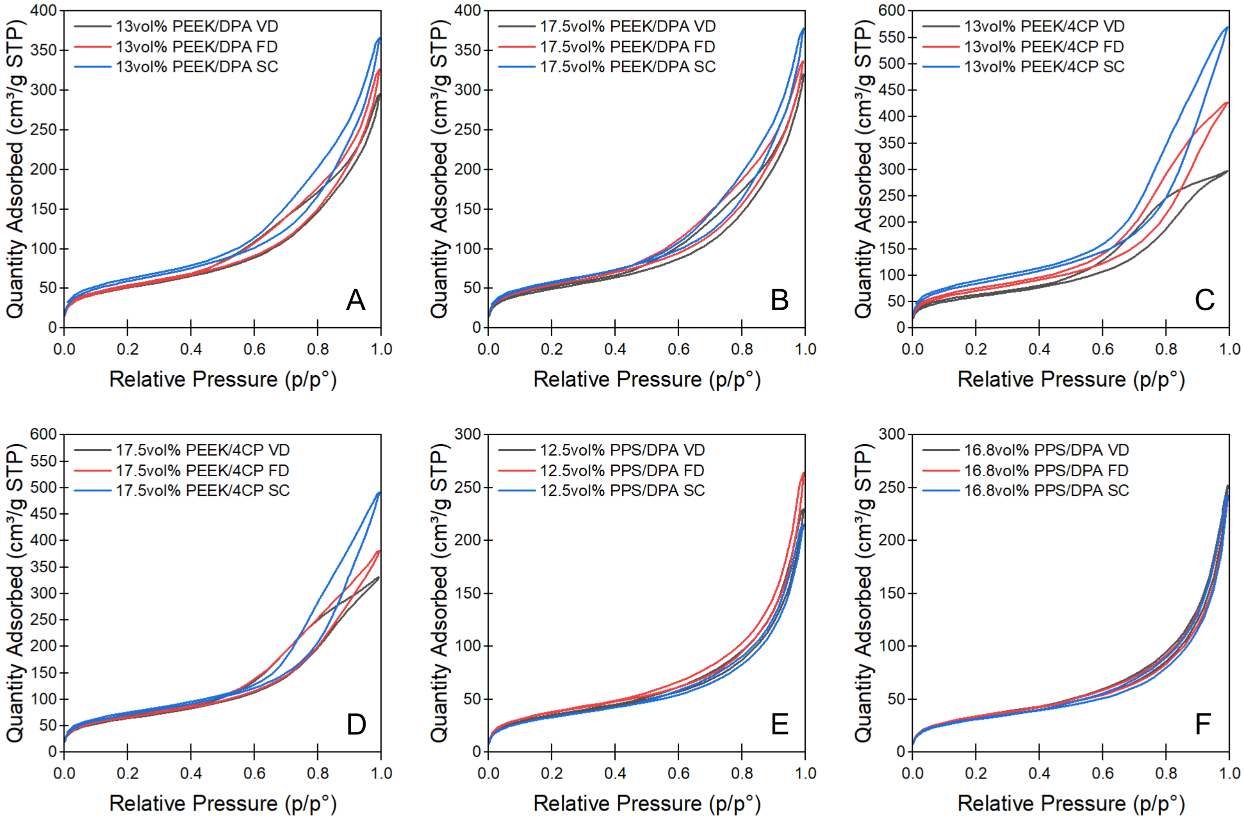

Figure S13. Nitrogen sorption isotherms of aerogels prepared from solution compositions of (a) 13.0vol% PEEK/DPA, (b) 17.5vol% PEEK/DPA, (c) 13.0vol% PEEK/4CP, (d) 17.5vol% PEEK/4CP, (e) 12.5vol% PPS/DPA, and (f) 16.8vol% PPS/DPA.

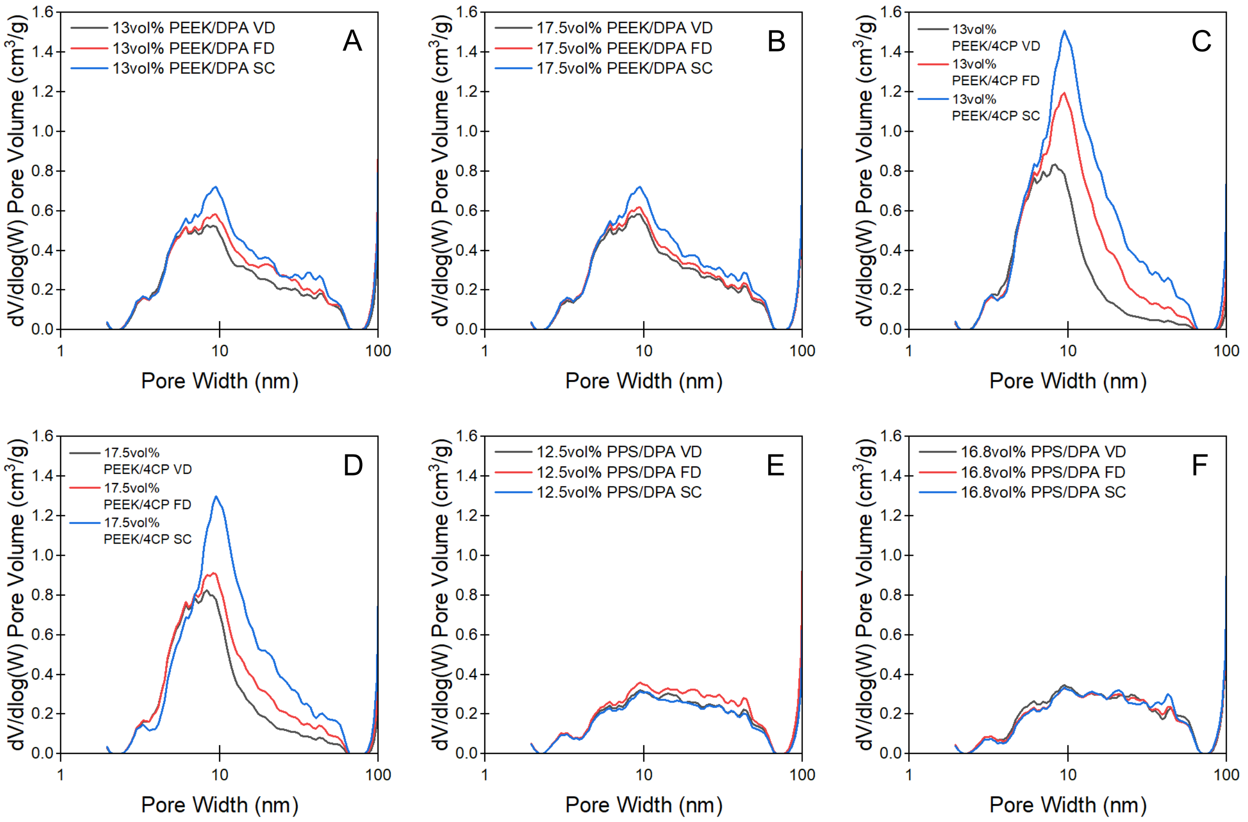

Figure S14. Pore size distribution of aerogels prepared from solution compositions of (a) 13.0vol% PEEK/DPA, (b) 17.5vol% PEEK/DPA, (c) 13.0vol% PEEK/4CP, (d) 17.5vol% PEEK/4CP, (e) 12.5vol% PPS/DPA, and (f) 16.8vol% PPS/DPA.

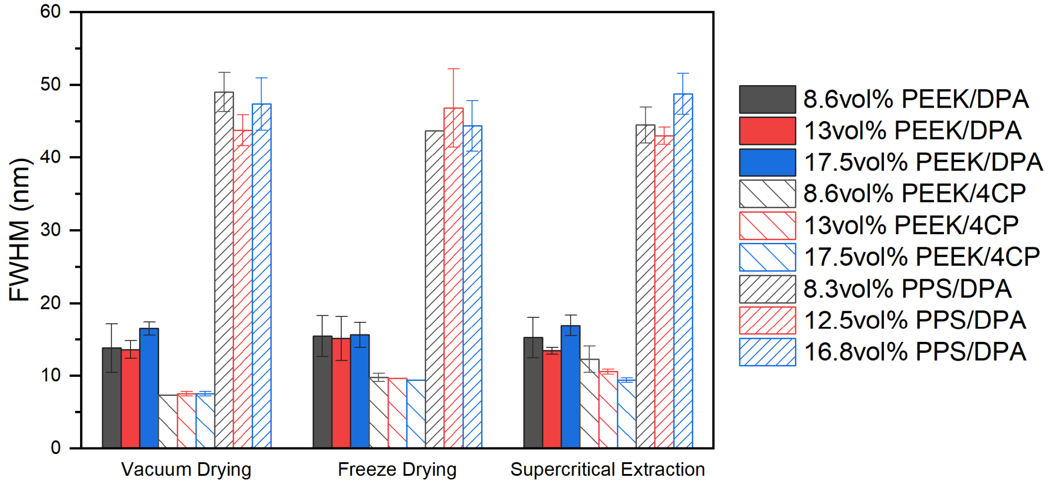

Figure S15. Peak full width at half maximum (FWHM) of pore size distributions.

Pore volume analysis

NLDFT analysis also yields the specific mesopore volume. This allows evaluation of the total porosity by pore size. Specific pore volume, which includes all pore sizes, for aerogels made in this study can be seen in **Figure S16**. A plot containing the specific pore volume for each aerogel system can be seen in **Figure S17a**. Tabulated pore properties, including specific pore volume, can be seen in **Table S3**. For all aerogel systems, total pore volume tends to decrease with increasing polymer content. The vacuum dried 8.3vol% PPS aerogels are an outlier, as they shrink excessively and have a low pore volume as a consequence. With increasing polymer content, more of the open aerogel porosity is occupied with the polymer network. Aerogels prepared with drying methods that cause the most shrinkage, vacuum drying and freeze drying, tend to have lower pore volumes than aerogels prepared with supercritical drying. Vacuum dried samples tend to have considerably lower pore volume than freeze dried samples, which are just slightly lower than samples prepared with supercritical extraction. Aerogels prepared from PEEK/4CP solutions also tend to have lower pore volumes compared to aerogels prepared from PEEK/DPA solutions (**Figure S17a**). Aerogels that shrink more show losses in pore volume, as empty volume is filled with the PEEK network during shrinkage. PPS aerogels tend to have slightly lower specific pore volume than PEEK/DPA aerogels.

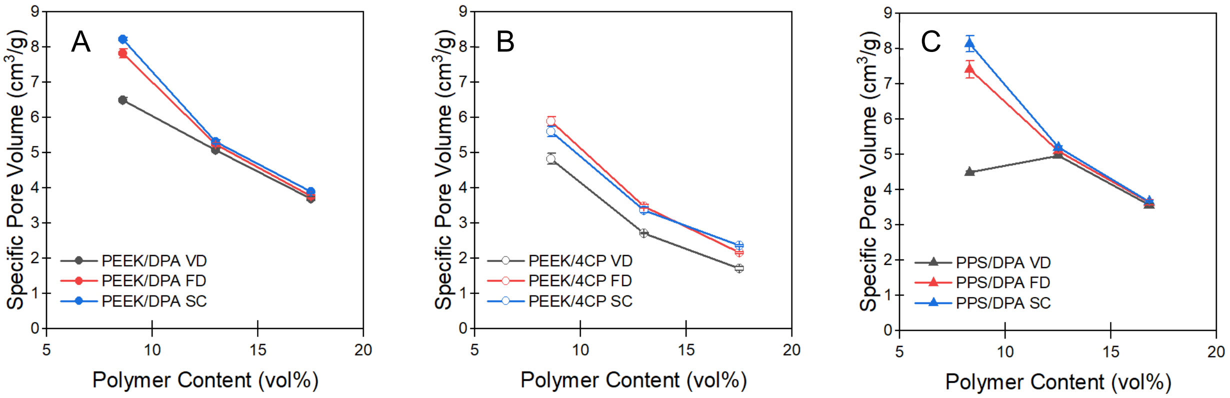

Figure S16. Specific pore volume of aerogels prepared from (a) PEEK/DPA solutions, (b) PEEK/4CP solutions, and (c) PPS/DPA solutions.

Specific mesopore volume for aerogels can be seen in **Figure S18**. A plot of the specific mesopore volume for each aerogel system can be seen in **Figure S17b**. Tabulated values for specific mesopore volume can be found in **Table S3**. Specific mesopore volume for PEEK aerogels prepared from DPA can be seen in **Figure 18a**. Mesopore volume does not appear to trend with polymer content for freeze dried or supercritially dried samples, but does appear to weakly increase with increasing polymer content for vacuum dried samples. Specific mesopore volume is not expected to change strongly with increasing polymer content, as the polymer aggregates, axialites, in the case of PEEK/DPA aerogels, host the mesopores within their surface texture. Mesopore volume is highest for PEEK/DPA aerogels prepared with supercritical extraction, lower for aerogels prepared with freeze drying, and lowest for aerogels prepared with vacuum drying. These findings are in great agreement with the SEM images (**Figure 2**), where the surface texture of the axialites is most affected by the drying method. The more aggressive drying methods of vacuum drying and freeze drying lead to larger forces during drying, which manifests as lower mesopore volumes.

Table S3. Aerogel pore properties: specific pore volume, specific mesopore volume, specific macropore volume, and mesopore volume fraction.

Gel System	Drying method	Specific Pore Volume (cm^3/g)	Specific Mesopore Volume (cm^3/g)	Specific Macropore Volume (cm^3/g)	Mesopore Volume Fraction (%)
8.6vol% PEEK/DPA	Vacuum Drying	6.50 ± 0.07	0.403 ± 0.035	6.10 ± 0.08	6.2 ± 0.5
8.6vol% PEEK/DPA	Freeze Drying	7.82 ± 0.13	0.460 ± 0.058	7.36 ± 0.14	5.9 ± 0.7
8.6vol% PEEK/DPA	Supercritical Extraction	8.23 ± 0.05	0.526 ± 0.048	7.71 ± 0.07	6.4 ± 0.6
13vol% PEEK/DPA	Vacuum Drying	5.07 ± 0.06	0.434 ± 0.015	4.63 ± 0.06	8.6 ± 0.3
13vol% PEEK/DPA	Freeze Drying	5.24 ± 0.04	0.481 ± 0.015	4.76 ± 0.04	9.2 ± 0.3
13vol% PEEK/DPA	Supercritical Extraction	5.32 ± 0.05	0.538 ± 0.021	4.78 ± 0.06	10.1 ± 0.4
17.5vol% PEEK/DPA	Vacuum Drying	3.70 ± 0.05	0.461 ± 0.005	3.24 ± 0.06	12.4 ± 0.2
17.5vol% PEEK/DPA	Freeze Drying	3.76 ± 0.05	0.481 ± 0.018	3.28 ± 0.05	12.8 ± 0.5
17.5vol% PEEK/DPA	Supercritical Extraction	3.89 ± 0.03	0.535 ± 0.018	3.35 ± 0.03	13.8 ± 0.5
8.6vol% PEEK/4CP	Vacuum Drying	4.83 ± 0.15	0.484 ± 0.005	4.34 ± 0.15	10.0 ± 0.3
8.6vol% PEEK/4CP	Freeze Drying	5.89 ± 0.13	0.722 ± 0.022	5.17 ± 0.13	12.3 ± 0.5
8.6vol% PEEK/4CP	Supercritical Extraction	5.61 ± 0.14	0.890 ± 0.027	4.72 ± 0.15	15.9 ± 0.6
13vol% PEEK/4CP	Vacuum Drying	2.71 ± 0.02	0.455 ± 0.022	2.26 ± 0.03	16.8 ± 0.8
13vol% PEEK/4CP	Freeze Drying	3.48 ± 0.06	0.635 ± 0.026	2.84 ± 0.06	18.3 ± 0.8
13vol% PEEK/4CP	Supercritical Extraction	3.37 ± 0.08	0.815 ± 0.034	2.56 ± 0.09	24.2 ± 1.1
17.5vol% PEEK/4CP	Vacuum Drying	1.71 ± 0.05	0.481 ± 0.009	1.23 ± 0.05	28.0 ± 1.0
17.5vol% PEEK/4CP	Freeze Drying	2.16 ± 0.04	0.548 ± 0.017	1.61 ± 0.04	25.4 ± 0.9
17.5vol% PEEK/4CP	Supercritical Extraction	2.36 ± 0.03	0.815 ± 0.012	1.55 ± 0.04	34.5 ± 0.7
8.3vol% PPS/DPA	Vacuum Drying	4.49 ± 0.03	0.341 ± 0.016	4.15 ± 0.04	7.6 ± 0.4
8.3vol% PPS/DPA	Freeze Drying	7.41 ± 0.25	0.323 ± 0.017	7.09 ± 0.25	4.4 ± 0.3
8.3vol% PPS/DPA	Supercritical Extraction	8.14 ± 0.23	0.326 ± 0.006	7.82 ± 0.23	4.0 ± 0.1
12.5vol% PPS/DPA	Vacuum Drying	4.98 ± 0.02	0.326 ± 0.015	4.65 ± 0.03	6.5 ± 0.3
12.5vol% PPS/DPA	Freeze Drying	5.10 ± 0.06	0.343 ± 0.014	4.76 ± 0.06	6.7 ± 0.3
12.5vol% PPS/DPA	Supercritical Extraction	5.20 ± 0.04	0.319 ± 0.006	4.88 ± 0.04	6.1 ± 0.1
16.8vol% PPS/DPA	Vacuum Drying	3.57 ± 0.04	0.335 ± 0.018	3.23 ± 0.04	9.4 ± 0.5
16.8vol% PPS/DPA	Freeze Drying	3.64 ± 0.07	0.346 ± 0.005	3.29 ± 0.07	9.5 ± 0.2
16.8vol% PPS/DPA	Supercritical Extraction	3.66 ± 0.03	0.343 ± 0.003	3.32 ± 0.03	9.4 ± 0.1

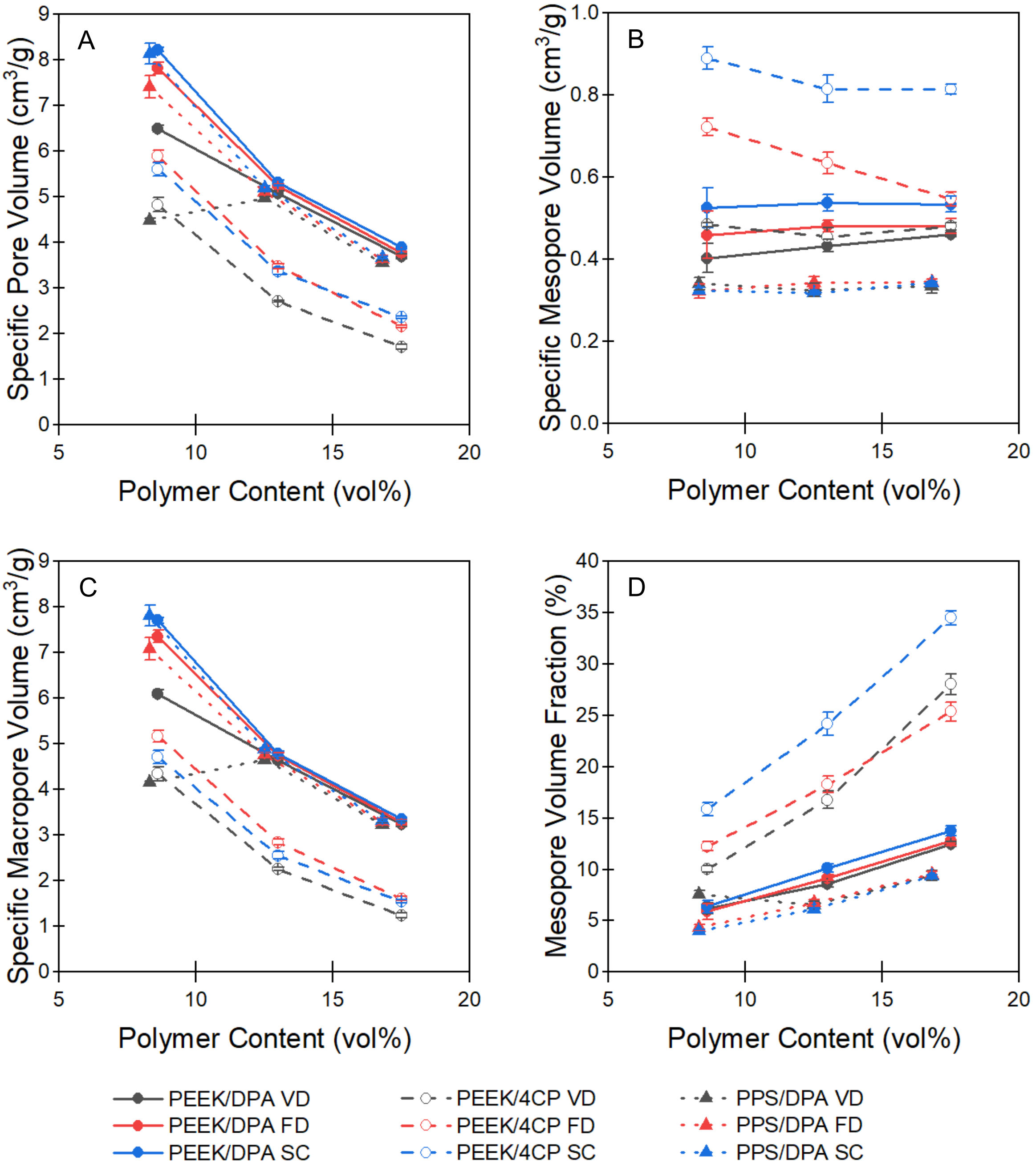

Figure S17. Aerogel pore properties: (a) specific pore volume, (b) specific mesopore volume, (c) specific macropore volume, and (d) mesopore volume fraction.

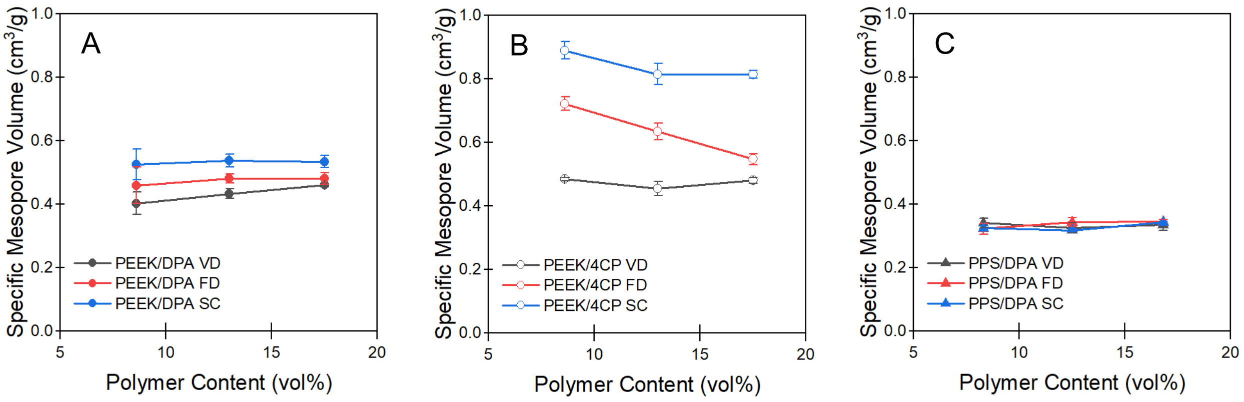

Figure S18. Specific mesopore volume of aerogels prepared from (a) PEEK/DPA solutions, (b) PEEK/4CP solutions, and (c) PPS/DPA solutions.

Specific mesopore volume for PEEK aerogels prepared from 4CP solutions can be found in **Figure 18b**. Mesopore volume does not appear to trend with polymer content for vacuum dried or supercritially dried samples, but does appear to decrease with increasing polymer content for freeze dried samples. Mesopore volume is highest for samples prepared by supercritical extraction, followed by samples prepared by freeze drying, then followed by vacuum dried samples, with the lowest mesopore volume. Mesopore volume is considerably higher, up to 0.9 m^2/g, for samples prepared from 4CP solutions dried with freeze drying or supercritical extraction. Vacuum drying considerably decreases the volume of mesopores present in aerogels prepared from PEEK/4CP. Drying method has a larger effect on mesopore volume of PEEK/4CP aerogels compared to PEEK/DPA aerogels. Vacuum drying of samples prepared from DPA or 4CP solutions results in a comparable range of mesopore volumes, but freeze drying or supercritical extraction of PEEK/4CP aerogels results in considerably higher mesopore volumes than is observed for PEEK/4CP aerogels prepared with vacuum drying. The smaller feature size of PEEK aerogels prepared from 4CP

(**Figure 3**) are believed to host more mesoporosity, with a higher surface area, opposed to the larger features observed in PEEK aerogels prepared from DPA (**Figure 2**).

Specific mesopore volume for PPS aerogels prepared from DPA can be seen in **Figure 18c**. For PPS aerogels, neither polymer content nor drying method had an effect on mesopore volume. All PPS aerogels displayed mesopore volumes around 0.3 cm^3/g. PPS aerogels tend to have lower mesopore volume than either of the PEEK aerogel systems (**Figure S17b**). The struts that compose PEEK aerogels prepared from DPA appear to have more surface texture (**Figure 2**) than the fibrils that compose PPS aerogels do (**Figure 4**).

Specific macropore volume for aerogels prepared from PEEK/DPA, PEEK/4CP, or PPS/DPA solutions can be seen in **Figure S19a**, **S19b**, **or S19c**, respectively. A comparison of the macropore volume for each system can be seen in **Figure S17c**. Tabulated values for specific macropore volume can be seen in **Table S3**. Generally, the same trends observed for specific pore volume are observed for specific macropore volume, since mesopore volume is considerably lower than macropore volume. Macropore volume decreases with increasing polymer content for all samples, except for the PPS aerogel prepared from 8.3 vol% PPS/DPA solution, which shrank considerably. Increasing the polymer content fills more of the macropore volume with the polymer network, leading to a decrease in microporosity. For PEEK or PPS aerogels prepared from DPA, macropore volume is strongly affected by drying method for gels prepared at 8.6 vol% PEEK or 8.3 vol% PPS. For aerogels prepared from DPA at higher polymer content, macropore content is not affected by drying method.

Interestingly, for the PEEK/4CP system, freeze drying resulted in a higher macropore volume than drying with supercritical extraction. The ability of a liquid to freeze within a pore is related to the pore size, with liquid contained in smaller pores requiring large undercoolings to

freeze.[4] Without sufficient undercoolings, liquid will not freeze within small, nanometer-scale pores of a gel, but can crystallize in the larger macropores. The crystal within a macropore exerts a force on the gel matrix, and if the gel does not have the strength to resist it, the crystal will continue growing in the macropore through expanding and deforming the matrix.[4] For globular PEEK aerogels prepared from 4CP the network is weaker than the aerogels with a strutlike network and is less able to resist the forces of crystal growth on the network. As ice crystals grow, they push the weak, globular network out of the way, thereby increasing macropore volume. The smaller mesopores still shrink in this system since fluid filling the pores tends to migrate to crystal growth front rather than crystallizing within small pores.

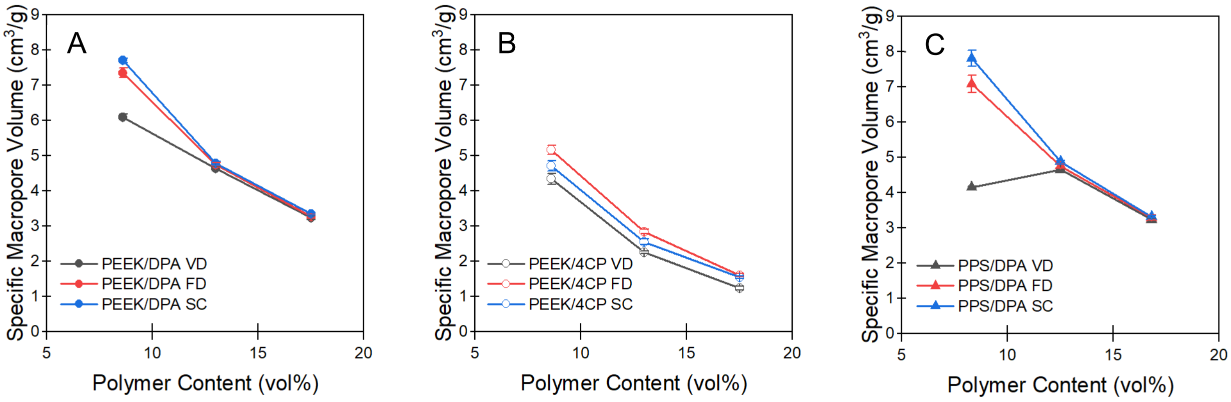

Figure S19. Specific macropore volume of aerogels prepared from (a) PEEK/DPA solutions, (b) PEEK/4CP solutions, and (c) PPS/DPA solutions.

Mesopore content of aerogels, expressed as a percentage of total porosity, can be seen in **Fig S20a-c**. A plot containing the mesopore content for each aerogel system can be seen in **Figure S17d**. Tabulated values for mesopore content can be found in **Table S3**. Generally, increasing polymer content tends to increase the mesopore fraction. The aerogel prepared from an 8.3 vol% PPS/DPA solution does not follow this trend, as it shrinks considerably. Increasing polymer content

replaces empty volume, once occupied with macropores, with semicrystalline polymer aggregates, which are covered with surface features that give rise to mesopores. With increasing the aggressiveness of the drying method, from supercritical drying to freeze drying to vacuum drying, the mesopore fraction tends to decrease. This dependence on the drying method is subtle for aerogels prepared from PEEK/DPA or PPS/DPA solutions but is considerable for aerogels prepared from PEEK/4CP solutions. Aerogels that shrink more, namely the vacuum dried aerogels prepared at either low polymer contents for PEEK/DPA and PPS/DPA systems, or at higher polymer contents for PEEK/4CP systems tend to have higher mesopore fractions due to the decrease in macropore volume due to shrinkage. Aerogels prepared from PEEK/4CP are more sensitive to changes induced during drying due to their weaker mechanical properties. Aerogels prepared from PEEK/DPA or PPS/DPA solutions have lower mesopore fractions than the aerogels prepared from PEEK/4CP solutions. With a smaller feature size and higher surface area, the aerogels prepared from PEEK/4CP may have a higher likelihood of containing mesopores.

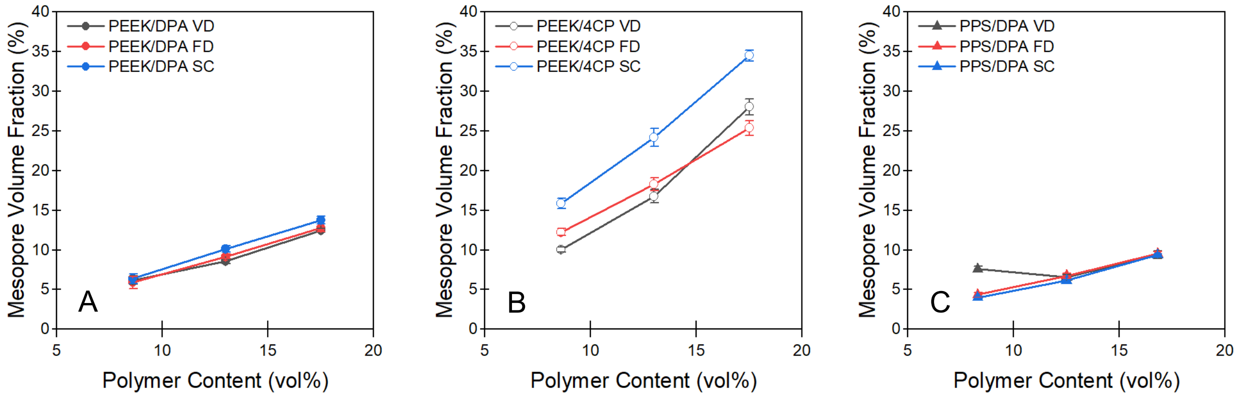

Figure S20. Mesopore volume fraction of aerogels prepared from (a) PEEK/DPA solutions, (b) PEEK/4CP solutions, and (c) PPS/DPA solutions.

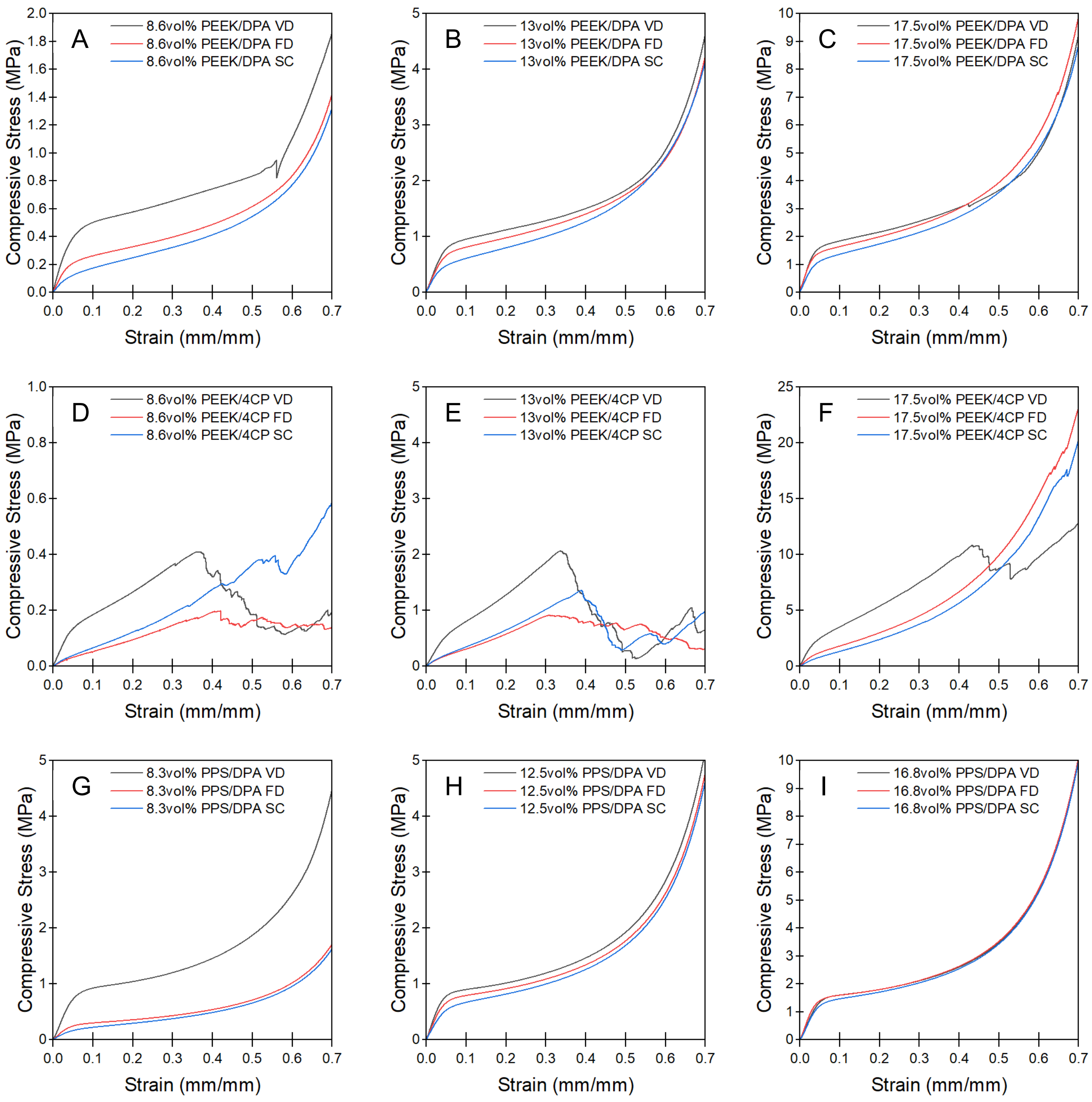

Figure S21. Compressive stress-strain curves for aerogels prepared from (a) an 8.6 vol.% PEEK/DPA solution, (b) a 13 vol.% PEEK/DPA solution, (c) a 17.5 vol.% PEEK/DPA solution, (d) an 8.6 vol.% PEEK/4CP solution, (e) a 13 vol.% PEEK/4CP solution, (f) a 17.5 vol.% PEEK/4CP solution, (g) an 8.3 vol.% PPS/DPA solution, (h) a 12.5 vol.% PPS/DPA solution, or (i) a 16.8 vol.% PPS/DPA solution, dried with different drying methods.

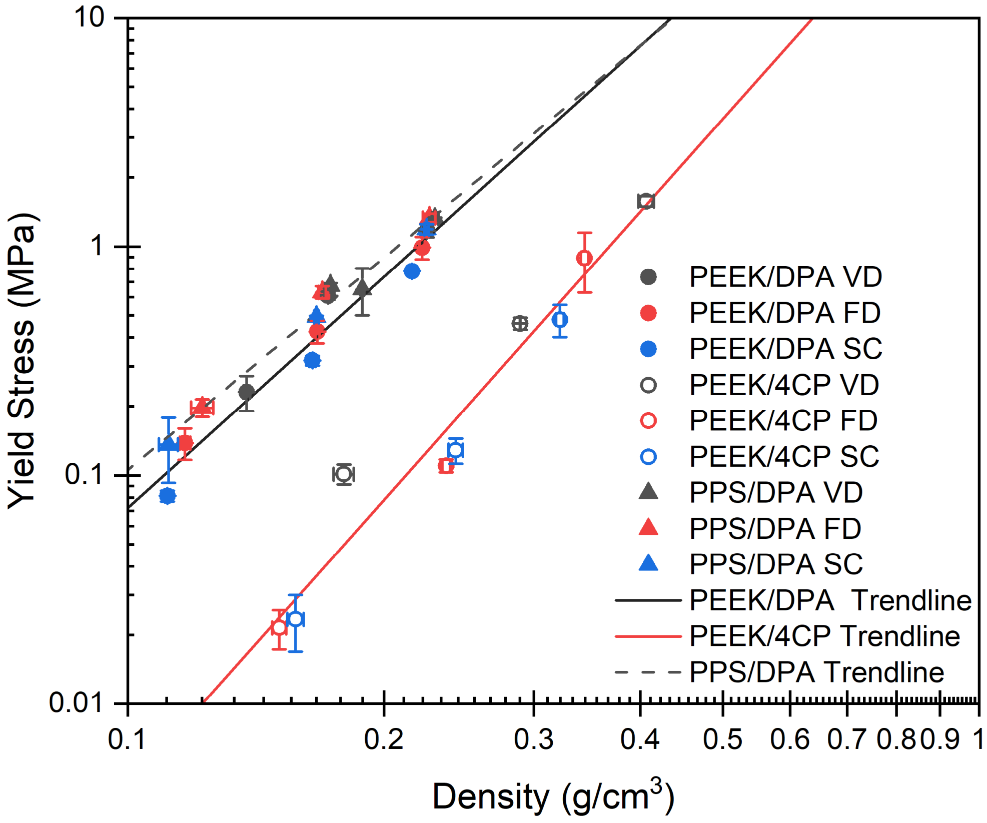

Figure S22. Offset yield stress vs aerogel density for polymer aerogels.

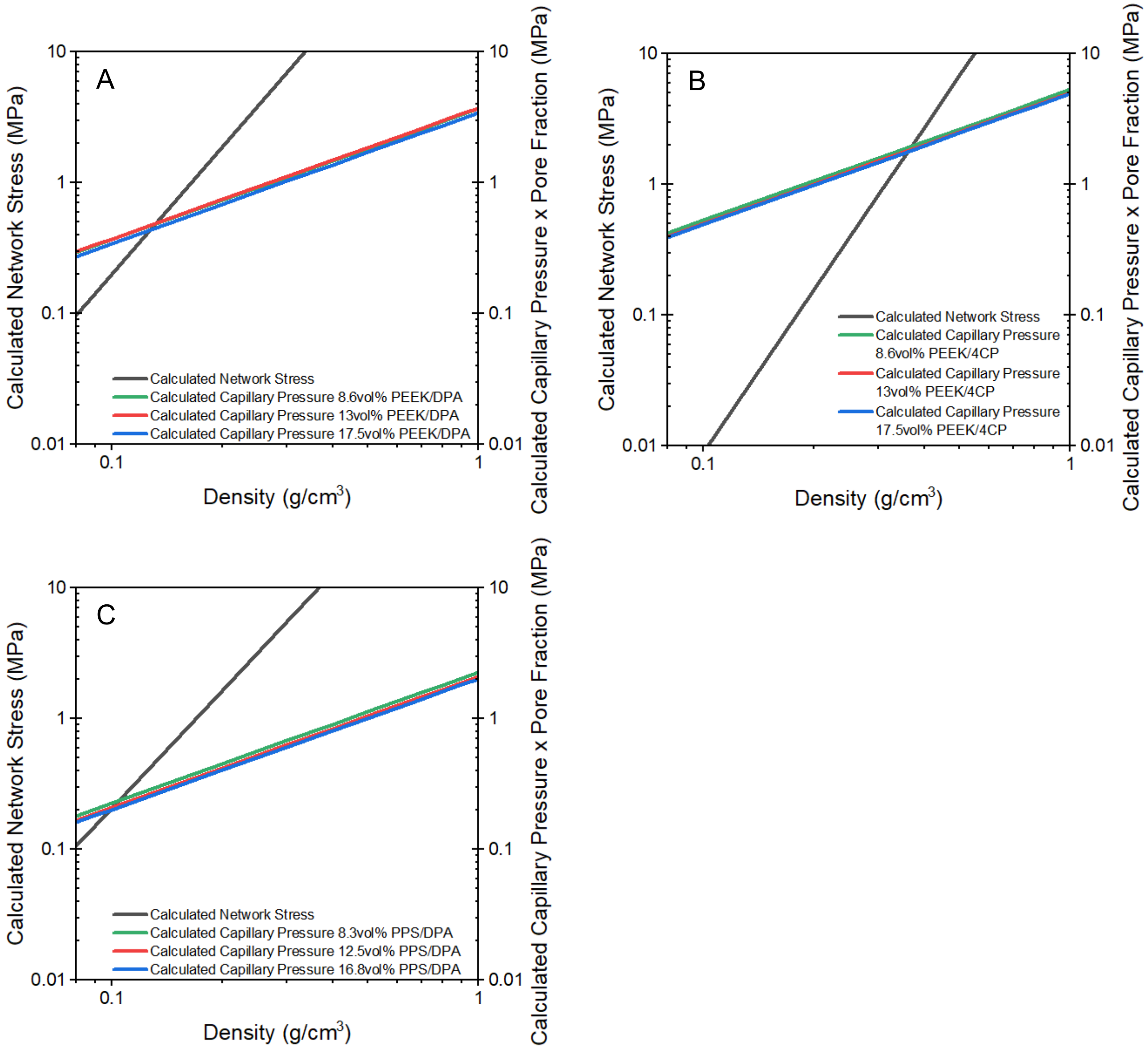

Figure S23. Network stress and capillary pressure vs density for (a) PEEK/DPA, (b) PEEK/4CP, or (c) PPS/DPA aerogels.

Supporting Information References

1. Beaucage, G. Approximations leading to a unified exponential/power-law approach to small-angle scattering. *J. Appl. Crystallogr.* **1995,** *28* (6), 717-728.

2. Roe, R.-J. *Methods of X-ray and neutron scattering in polymer science*. Oxford University Press on Demand: 2000.

3. Smith, D.; Scherer, G.; Anderson, J. Shrinkage during drying of silica gel. *Journal of Non-Crystalline Solids* **1995,** *188* (3), 191-206.

4. Scherer, G. W. Freezing gels. *Journal of Non-Crystalline Solids* **1993,** *155* (1), 1-25.
